# Male fertility in *Arabidopsis* requires active DNA demethylation of genes that control pollen tube function

**DOI:** 10.1038/s41467-020-20606-1

**Published:** 2021-01-18

**Authors:** Souraya Khouider, Filipe Borges, Chantal LeBlanc, Alexander Ungru, Arp Schnittger, Robert Martienssen, Vincent Colot, Daniel Bouyer

**Affiliations:** 1 Institut de Biologie de l’Ecole Normale Supérieure (IBENS), Centre National de la Recherche Scientifique (CNRS), Institut National de la Santé et de la Recherche Médicale (INSERM), Ecole Normale Supérieure, PSL Research University, 75005 Paris, France; 2 Howard Hughes Medical Institute, Cold Spring Harbor Laboratory, Cold Spring Harbor, New York, NY 11724 USA; 3 Institut Jean-Pierre Bourgin, INRAE, AgroParisTech, Université Paris-Saclay, 78000 Versailles, France; 4 Max Planck Institute for Plant Breeding Research, 50829 Cologne, Germany; 5 Institut de Biologie Moleculaire des Plantes (IBMP), CNRS, University Strasbourg, 67084 Strasbourg, France; 6 Present Address: Faculty of Arts and Sciences, Department of Molecular, Cellular and Developmental Biology, Yale University, New Haven, CT 06511 USA; 7 Present Address: University Hamburg, 22609 Hamburg, Germany; 8 Present Address: Laboratoire Reproduction et Développement des Plantes (RDP), UnivLyon, Ecole Normale Supérieure de Lyon, Université Claude Bernard Lyon1, CNRS, INRAE, 69342 Lyon, France

**Keywords:** DNA methylation, Plant embryogenesis, Fertilization

## Abstract

Active DNA demethylation is required for sexual reproduction in plants but the molecular determinants underlying this epigenetic control are not known. Here, we show in *Arabidopsis thaliana* that the DNA glycosylases DEMETER (DME) and REPRESSOR OF SILENCING 1 (ROS1) act semi-redundantly in the vegetative cell of pollen to demethylate DNA and ensure proper pollen tube progression. Moreover, we identify six pollen-specific genes with increased DNA methylation as well as reduced expression in *dme* and *dme;ros1*. We further show that for four of these genes, reinstalling their expression individually in mutant pollen is sufficient to improve male fertility. Our findings demonstrate an essential role of active DNA demethylation in regulating genes involved in pollen function.

## Abstract (web summary)

Active DNA demethylation is required for sexual reproduction in plants, but the underlying mechanisms are unknown. Here, the authors show that the DNA glycosylases DEMETER and REPRESSOR OF SILENCING 1 enable the DNA demethylation-dependent activation of genes involved in pollen tube progression.

## Introduction

DNA cytosine methylation (5-Methylcytosine) is an epigenetic mark that plays critical roles in the silencing of transposable elements (TEs) and the transcriptional regulation of genes in plants and mammals^1–3^. In plants, methylation affects cytosines in the three possible contexts, CG, CHG, and CHH (where H = A, C, or T) through different molecular pathways and DNA methyltransferases (MTases). In *Arabidopsis thaliana*, DRM1 and DRM2, which are related to the mammalian de novo DNA MTase Dnmt3, establish DNA methylation in all sequence contexts in an RNA-dependent manner (RNA-directed DNA methylation or RdDM^4^). DRM1 and 2, together with CMT2, a plant-specific chromomethylase, are also essential for the maintenance of CHH methylation. In addition, MET1, which is the plant homolog of the maintenance DNA MTase Dnmt1 and the chromomethylase CMT3 are responsible for maintaining CG and CHG methylation, respectively^1,5^.

Although in mammals DNA methylation is reduced drastically in the early embryo and even more so in the primordial germlines, broad DNA demethylation has not been observed in the gametes or the embryo of plants, except in the CHH context^6–8^. In contrast, extensive DNA demethylation occurs in the so-called gamete companion cells that are part of the post-meiotic multicellular structures, the gametophytes, in which gametes of flowering plants are embedded. Specifically, pollen, which is the male gametophyte, contains a vegetative cell (VC), which is hypomethylated—mainly in the CG context—at specific genomic regions compared to the two sperm cells (SCs) that it encapsulates^9,10^. On the female side, the ovule includes two synergids, which play important roles for pollen tube attraction, the central cell (CC), which is globally hypomethylated—again mainly in the CG context—and the egg cell (EC)^11^.

In both the VC and CC, DNA demethylation mainly depends on the activity of the DNA glycosylase DEMETER (DME)^9^. Loss of DME leads to complete seed abortion when maternally inherited^12^, possibly as a result of defects in genomic imprinting^13^. On the paternal side, loss of DME leads to partial sterility, the degree of which varies between genetic backgrounds^14^. DNA demethylation in the VC correlates with the reactivation of TEs and the production of small RNAs (sRNAs), which may be transported to the two SCs. Based on these observations, it was proposed that demethylation in the VC serves to reinforce silencing of TEs, via sRNAs, in the SCs^9,10,15,16^. However, few TEs are reactivated in VCs^17^ and it is unclear how defects in TE reactivation in DME-deficient pollen would cause male sterility. Thus, an alternative possibility is that active DNA demethylation is required in the VC to enable the expression of genes involved in pollen function^14,17^.

To address this possibility, we analyze pollen defective in DNA demethylase activity. We extend our analyses to situations where both *DME* and *ROS1*, which encodes the main DNA demethylase in somatic tissues^18,19^, are mutated. Our findings indicate that ROS1 acts together with DME in the VC to ensure proper genome-wide DNA demethylation, as well as proper activation of a small number of genes that define a signaling complex critical for pollen tube function.

## Results

### DME and ROS1 act together to ensure male fertility

**Fig. 1:**
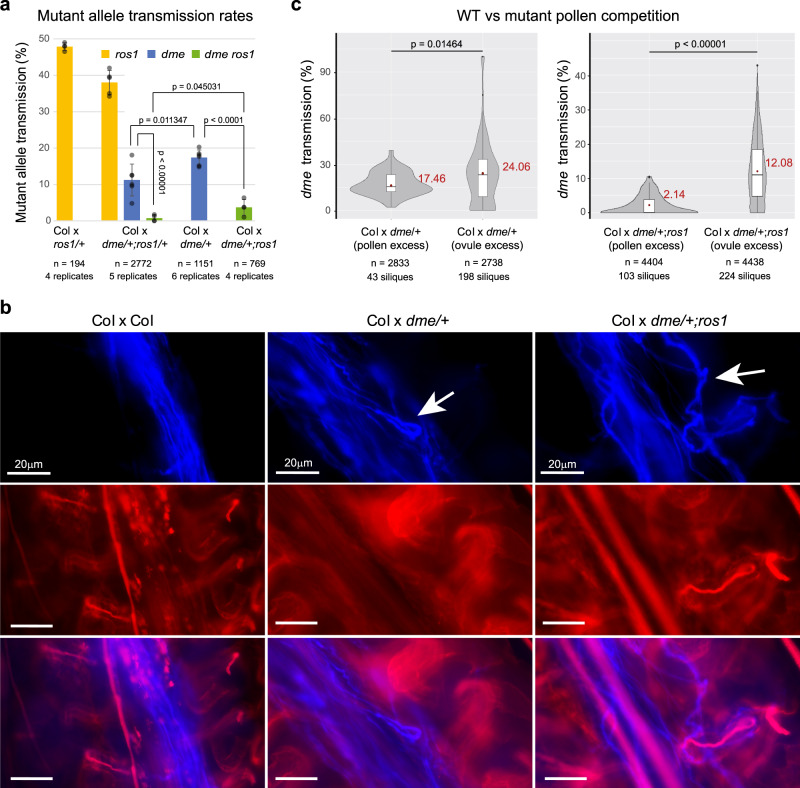
DME and ROS1 act semi-redundantly to promote pollen tube progression. **a** Transmission frequency of the *dme* and *ros1* alleles in crosses between Col-0 wild-type mothers and *ros1/+*, *dme/+*, *dme/+;ros1/+*, and *dme/+;ros1* pollen donors as bar plot giving the mean ± SD as error bars and individual data points shown as gray dots. Number of individual plants and biological replicates is given below the graph and pairwise comparisons of *dme* transmission rate between the different crosses revealed significant differences (*p*-values of two-sided *T*-tests in pairwise comparison is shown in the graph). **b** Fluorescent microscopy of aniline blue stained pistils 24 h after crosses of wild-type Col-0 mothers with Col-0, *dme/+* and *dme/+;ros1* fathers, respectively, revealing pollen tubes in the transmitting tract. Red channel shows autofluorescence of maternal tissues, including the ovules. White arrow indicates aberrant pollen tube trajectory in the mutants; white scale bar equals 20 µm in all photographs. **c** Violin plots showing the distribution of the transmission frequency of the *dme* allele in individual siliques from crosses between wild-type Col-0 mother plants and *dme/+*, as well as *dme/+;ros1* fathers with either excess of pollen or excess of ovules (few pollen grains used in the respective cross). Inserted boxplots represent 50% of all values with the second and third quartile separated by the median value as black line, mean value is shown as a red rhomb together with the number (in red). Number of individual plants and siliques (which equals replicates) as well as *p*-value of two-sided *T*-test is given. Source data underlying Fig. 1a, c are provided as a Source Data file.

We first measured the transmission rate of the *dme* mutant allele in crosses with wild-type mothers of the reference strain Columbia (Col-0). Fathers used in these crosses were heterozygous for *dme* (*dme/+*, and either wild type or homozygous mutant for *ROS1* (the latter designated *dme/+;ros1*). Consistent with previous observations^14^, transmission of the *dme* mutant allele was reduced to 17.4% instead of the 50% expected in the case of Mendelian segregation at a single locus (Fig. 1a). In addition, we found that *dme* transmission was much further reduced when the *dme/+;ros1* double mutant was used as pollen donor (3.2% vs. 50%; Fig. 1a), suggesting that ROS1 activity compensates partially for the lack of DME. Crosses involving double heterozygous *dme/+;ros1/+* fathers confirmed these results, with a reduced transmission of *dme* to 11.4% when alone and to only 1.4% when combined with *ros1*, compared to 25% expected under Mendelian segregation in both cases. In contrast, transmission of *ros1* alone (i.e., in combination with the *DME* wild-type allele) was not affected (Fig. 1a). Together, these results indicate that DME and ROS1 have partially redundant functions in male fertility, with DME playing a more prominent role.

### DME and ROS1 are both required for proper pollen tube orientation

To identify the origin of the transmission deficiency of *dme*, we first examined the possibility of a meiotic defect. To this end we made use of the Fluorescent Tagged Line, FTL_1311, which harbors a transgene encoding a pollen-specific fluorescent marker as well as the *quartet* (*qrt*) mutation^20^. This mutation causes failure of microspore separation during pollen development, hence providing an easy means to verify the integrity of the four haploid cells that result from meiosis. The transgene present in line FTL_1311 is closely linked (973 kb and 1.5 cM) to the *DME* locus (Supplementary Fig. 1a) and we first introgressed it together with the *qrt* mutant allele in the hemizygous state into the *dme/+* and *dme/+;ros1* backgrounds. We then determined the proportion of fluorescent pollen in the resulting lines, which should reflect the proportion of *DME* wild-type pollen given the tight linkage of the transgene to the *DME* wild-type allele (Supplementary Fig. 1a, b). The tetrads were intact and had two fluorescent pollen (Supplementary Fig. 1b), indicating no loss of the *dme* mutant allele through meiotic drive. In addition, pollen viability was not affected in the single mutant, consistent with previous observations^14^, nor in *dme/+;ros1* (Supplementary Fig. 1c). As pollen germination deficiency can only explain part of the *dme* transmission defect^14^, we next investigated if pollen tube elongation is impaired in *dme/+* and *dme/+;ros1*. Were this the case, we would expect less seeds carrying the *dme* allele in the distal part of the silique, as pollen tubes need to grow further to reach it. However, we did not detect significant differences in *dme* transmission rates between the upper and the lower part of siliques (Supplementary Fig. 1d), confirming previous observations made in *dme* single mutants^14^ and indicating that pollen tube growth per se is not reduced in *dme/+* or *dme/+;ros1*. We also did not observe any post-fertilization defects such as seed abortion in crosses with *dme/+*, consistent with a previous report^12^, nor *dme/+;ros1*-derived pollen (Supplementary Fig. 1e). Rather, many pollen tubes followed aberrant trajectories in the transmitting tract of the pistil in crosses involving mutant fathers (Fig. 1b). Closer examination suggested that *dme* and *dme;ros1* pollen tubes fail to orient properly in the maternal transmitting tract and thus rarely reach ovules before wild-type pollen. To determine whether *dme* pollen is indeed outcompeted by wild-type pollen, we applied limited amount of pollen grains to emasculated flowers, thereby providing an excess of ovules. As predicted, *dme* transmission was ameliorated under these conditions and increased significantly from 17% to 24% and from 2% to 12% in crossed with dme/+ and *dme/+;ros1* fathers, respectively (Fig. 1c). However, mutant pollen remains less efficient compared to wild-type pollen at fertilizing ovules, irrespective of the number of ovules fertilized per silique (Supplementary Fig. 1f). Taken together, these observations suggest that DME and to a lower extent ROS1 are required for the proper journey of the pollen tube towards the ovule.

### DME and ROS1 together are responsible for DNA demethylation of the VC

**Fig. 2:**
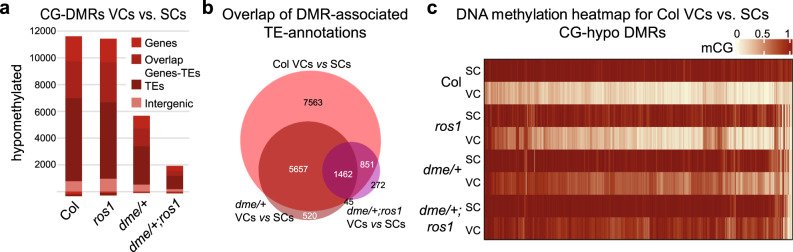
DME and ROS1 are responsible for DNA demethylation of the vegetative cell in pollen. **a** Number and association with genome annotations of CG-DMRs detected in the comparison between VCs and SCs in wild-type (Col), *ros1*, *dme/+* and *dme/+;ros1* mutant plants (hypomethylated = lower DNA methylation level in VCs). **b** Venn diagram showing the overlap of CG-hypo DMRs detected between the VCs and the SCs of Col-0 wild-type, *dme/+*, and *dme/+;ros1*. **c** Heatmap of absolute CG methylation level in SCs and VCs of CG-hypo DMRs detected between VCs and SCs in Col-0 wild type (11,107 regions). The lines represent the indicated genotype for SCs and VCs, respectively. DMRs in the columns were sorted by complete linkage hierarchical clustering using Euclidean distance.

To establish how DNA demethylation carried out by DME and ROS1 may affect pollen tube function, we analyzed the methylome of fluorescence-activated cell sorted (FACS) VCs and SCs of wild-type, single, and double mutant plants using whole-genome sequencing of bisulfite-converted genomic DNA (Supplementary Fig. 2 and Supplementary Data 1). Consistent with previous results^9,10^, methylation in wild-type pollen is lower at many CG sites in the VCs compared to the SCs (CG-hypomethylated regions, CG-hypo DMRs (differentially methylated regions); Fig. 2a, b). A small number of predominantly short CG-hyper DMRs are also observed and these tend to exhibit also higher CHH and—to a lesser extent—CHG methylation in VCs compared to SCs (Supplementary Fig. 3a–c and Supplementary Data 2). Previous work suggests that this hypermethylation in wild-type VCs results from higher RdDM activity in the VC than in the SCs^15^. Pollen produced from single and double mutant plants showed large differences in CHG and especially CG, but not CHH methylation compared to wild-type pollen (Supplementary Fig. 3a–c). Specifically, the number and size of CG-hypo DMRs, and to a lesser extent CHG-hypo DMRs, between VCs and SCs is much reduced in mutants (Fig. 2a, b, Supplementary Fig. 3a, c, and Supplementary Data 2–5). As pollen from *dme/+* and *dme/+;ros1* mutant plants is an equal mix of *DME* wild type and *dme* mutants (Supplementary Fig. 1c), these results suggest that the absence of DNA demethylation at CG sites in mutant VCs was sufficient to preclude the detection in this background of CG-hypo DMRs in the VCs vs. SCs comparison.

In agreement with this explanation, CG-hypo DMRs detected in the wild type between VCs and SCs had much higher CG methylation in mutant than in wild-type VCs (Fig. 2c) and they correspond to a large extent to the CG-hypo DMRs that are detected in the comparison of VCs between wild-type and mutant plants (Supplementary Fig. 3e, f and Supplementary Data 2 and 6–8). Most CG-hypomethylation in wild-type VCs is primarily found over TEs. Moreover, protein coding genes that associate with CG-hypo DMRs between wild-type VCs and SCs overlap to about 60% with TEs within 500 bp up- and downstream of their transcription unit, in contrast to 22.5% genome-wide^21^ (Fig. 2a), and these genes showed Gene Ontology enrichments such as “pollen tube guidance,” “acceptance of pollen,” “recognition of pollen,” and “regulation of pollen tube growth” (Tables 1–3 and Supplementary Data 2 and 6–8). Finally, more than 1/3 of the CG-hypo DMRs detected between wild-type VCs and SCs are located within the 5′-region of the associated genes (Supplementary Fig. 3d). Together, these observations provide strong support for the proposition made previously that TE sequences contribute to reproduction-specific gene expression by providing transcription factor binding sites that become accessible in the endosperm, as well as in the VC thanks to their demethylation^17,22–24^.

**Table 1:** GO-term enrichment for genes associated with hypo-CG-DMRs detected between Col VC and SC.

Term	Count^a^	%^b^	*p*-Value^c^	Fold enrichment
Histone methylation	6	0.3	0.001	8.0
Regulation of proteolysis	9	0.5	0.001	4.0
Cell wall macromolecule catabolic process	7	0.4	0.005	4.3
SCF-dependent proteasomal ubiquitin-dependent protein catabolic process	19	1.0	0.005	2.1
Protein polyubiquitination	16	0.8	0.005	2.2
Response to oxidative stress	30	1.5	0.008	1.7
Proteasome-mediated ubiquitin-dependent protein catabolic process	27	1.4	0.010	1.7
Pollen tube guidance	7	0.4	0.011	3.6
Amino sugar metabolic process	5	0.3	0.012	5.4
Killing of cells of other organism	29	1.5	0.013	1.6
Protein ubiquitination involved in ubiquitin-dependent protein catabolic process	20	1.0	0.014	1.8
Defense response to fungus	42	2.2	0.015	1.5
Response to jasmonic acid	18	0.9	0.017	1.9

^a^Number of genes from list.

^b^Percentage of genes in this category/Total number of genes in the list.

^c^Fisher’s exact *p*-value; <0.05 is considered strongly enriched (only values <0.02 are shown).

**Table 2:** GO-term enrichment for genes associated with hypo-CG-DMRs detected between Col and *dme* VCs.

Term	Count^a^	%^b^	*p*-Value^c^	Fold enrichment
Protein phosphorylation	253	5.0	0.000000	1.6
Embryo development ending in seed dormancy	126	2.5	0.000002	1.5
DNA repair	60	1.2	0.000040	1.7
Intracellular protein transport	59	1.2	0.000067	1.6
Gene silencing by RNA	21	0.4	0.000083	2.5
SCF-dependent proteasomal ubiquitin-dependent protein catabolic process	47	0.9	0.000150	1.7
(1->3)-β-d-glucan biosynthetic process	9	0.2	0.000330	4.0
Protein import into nucleus. Docking	12	0.2	0.000490	3.1
Defense response to bacterium	73	1.4	0.001100	1.4
Galactolipid biosynthetic process	8	0.2	0.001200	3.9
Cell growth	18	0.4	0.001900	2.2
Protein-chromophore linkage	18	0.4	0.001900	2.2
Acceptance of pollen	8	0.2	0.002500	3.6
Chloroplast organization	37	0.7	0.003300	1.6
Covalent chromatin modification	28	0.6	0.004000	1.7
Protein autophosphorylation	39	0.8	0.004000	1.6
Maintenance of DNA methylation	9	0.2	0.004300	3.0
Response to cadmium ion	84	1.7	0.004500	1.3
Trichome morphogenesis	14	0.3	0.005600	2.2
Pollen germination	20	0.4	0.006700	1.9
Recognition of pollen	15	0.3	0.007800	2.1
Innate immune response	22	0.4	0.008000	1.8
Regulation of pollen tube growth	10	0.2	0.008700	2.6
Transmembrane transport	54	1.1	0.011000	1.4
Intracellular signal transduction	49	1.0	0.011000	1.4

^a^Number of genes from list.

^b^Percentage of genes in this category/Total number of genes in the list.

^c^Fisher’s exact *p*-value; <0.05 is considered strongly enriched (only values <0.02 are shown).

**Table 3:** GO-term enrichment for genes associated with hypo-CG-DMRs detected between Col and *dme;ros1* VCs.

Term	Count^a^	%^b^	*p*-Value^c^	Fold enrichment
Protein phosphorylation	306	4.1	0.00000	1.3
Defense response to fungus	162	2.2	0.00005	1.3
Actin filament organization	15	0.2	0.00006	2.8
Embryo development ending in seed dormancy	156	2.1	0.00029	1.3
Intracellular signal transduction	72	1.0	0.00053	1.4
Protein processing	19	0.3	0.00093	2.1
Gene silencing by RNA	23	0.3	0.00150	1.9
Telomere maintenance	12	0.2	0.00210	2.5
Chloroplast organization	49	0.7	0.00240	1.5
Killing of cells of other organism	99	1.3	0.00270	1.3
Trichome morphogenesis	18	0.2	0.00290	2.0
Actin filament-based movement	12	0.2	0.00380	2.4
(1->3)-β-d-glucan biosynthetic process	9	0.1	0.00410	2.8
Potassium ion transmembrane transport	18	0.2	0.00420	1.9
SCF-dependent proteasomal ubiquitin-dependent protein catabolic process	55	0.7	0.00470	1.4
Phosphatidylinositol dephosphorylation	11	0.1	0.00490	2.4
Cellulose biosynthetic process	22	0.3	0.00540	1.8
Regulation of cell shape	19	0.3	0.00650	1.8
Positive regulation of proteasomal ubiquitin-dependent protein catabolic process	20	0.3	0.00680	1.8
Regulation of membrane potential	15	0.2	0.00970	2.0
Signal transduction	142	1.9	0.01200	1.2
Cell death	20	0.3	0.01200	1.7
Protein autophosphorylation	49	0.7	0.01300	1.4
Arp2/3 complex-mediated actin nucleation	7	0.1	0.01400	2.9
Transmembrane transport	72	1.0	0.01400	1.3
Regulation of ion transmembrane transport	9	0.1	0.01500	2.4
DNA duplex unwinding	12	0.2	0.01600	2.1
Innate immune response	27	0.4	0.01900	1.5
Response to bacterium	38	0.5	0.01900	1.4
Glucosinolate catabolic process	15	0.2	0.01900	1.8
Pollen germination	24	0.3	0.02000	1.6
Histone lysine methylation	8	0.1	0.02000	2.5

^a^Number of genes from list.

^b^Percentage of genes in this category/Total number of genes in the list.

^c^Fisher’s exact *p*-value; <0.05 is considered strongly enriched (only values <0.02 are shown).

### DME and ROS1 are essential for expression of genes involved in pollen tube function

**Fig. 3:**
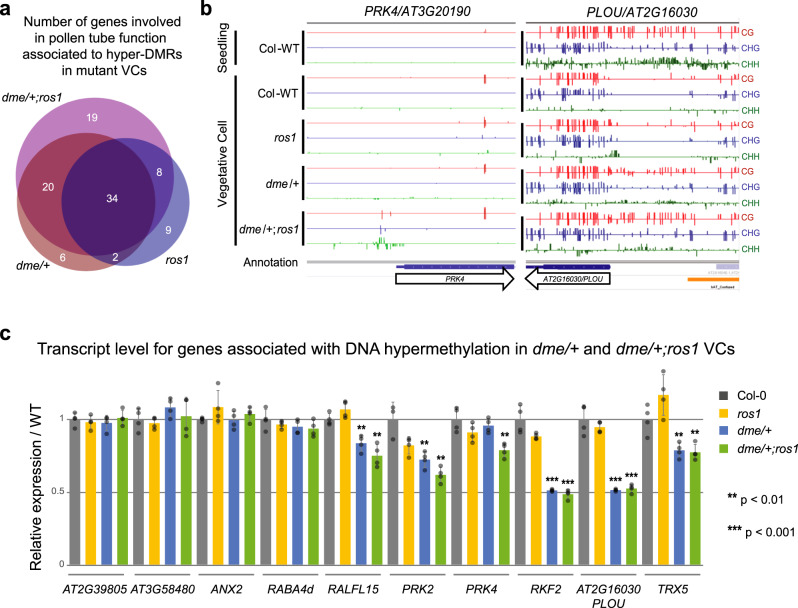
DME and ROS1 are required for the expression of genes involved in pollen tube function. **a** Venn diagram giving the number and overlap of genes implied in pollen tube function (see Supplementary Data 9) associated with hyper DMRs in the respective mutant VCs compared to wild-type VCs. **b** Genome-browser view of two genes involved in pollen tube function (*PRK4*/*AT3G20190* and *PLOU/AT2G16030*), showing the absolute DNA methylation level for each cytosine context as indicated in the graph for five samples (wild-type [Col-WT] seedlings and VCs of Col-0 wild type, *ros1*, *dme/+* and *dme/+;ros1*). **c** RT-qPCR from pollen of the given genotypes of transcripts showing lower abundance in *dme/+* and *dme/+;ros1* compared to wild-type (Col-0) pollen. For each sample four biological replicates have been used and relative expression level calculated with respect to the reference genes shown on the left. Bars represent mean values with SD shown as error bars. *p*-values of two-sided *T*-test in the respective comparison with wild-type level are indicated by stars above the bars; absence of stars indicates no significant difference (*p* > 0.05; see Supplementary Data 10 for exact *p*-values). Source data underlying Fig. 3c are provided as a Source Data file.

To investigate further the potential role of active DNA demethylation in pollen tube function, we established a list of 194 genes known or predicted to be involved in this function, based on literature research (see Supplementary Data 9). Remarkably, 108 of these genes showed VC DNA-hypomethylation, either in the comparison between wild-type VCs and SCs, and/or in the comparison between wild-type and mutant VCs (Fig. 3a and Supplementary Data 9). The gain of DNA methylation was most often over promoter regions not methylated in wild-type seedlings and pollen, except sometimes in SCs, the latter being in agreement with the observation of elevated RdDM activity in the male germline in *A. thaliana*^25^ (Fig. 3b, Supplementary Fig. 4 Class I, and Supplementary Data 9). In some cases, gain of DNA methylation in mutant VCs was over promoter regions, which were specifically demethylated in wild-type VCs (Fig. 3b, Supplementary Fig. 4 Class II, and Supplementary Data 9).

To determine whether this gain of DNA methylation over promoter sequences in mutant VCs was linked to transcriptional downregulation of the cognate genes, we measured transcript levels by reverse transcriptase quantitative PCR (RT-qPCR) for 46 genes that show ectopic DNA methylation over the upstream region (Supplementary Data 9). We did not take into account changes in gene body methylation, as these do not appear to affect expression^26^, nor those DMRs that are mainly dependent on *ros1* due to the absence of fertility defects in this mutant (Supplementary Data 9). As *dme/+* and *dme/+;ros1* mutants carry 50% *DME* wild-type pollen, we expected to observe at most a 50% reduction in transcript levels compared to wild type if promoter hypermethylation leads to full gene repression in mutant VCs. Six genes showed reduced transcript levels in mutant pollen, with repression being more pronounced and almost complete (half of the transcript level measured in wild type Col-0) in the double mutant for three genes (Fig. 3c and Supplementary Data 10). These six genes encode signaling components or are associated with receptor-like proteins^27–33^, which suggests a direct role for DME and ROS1 in enabling VC-specific expression of genes involved in pollen tube function.

### A pollen tube signaling network co-regulated by active DNA demethylation

**Fig. 4:**
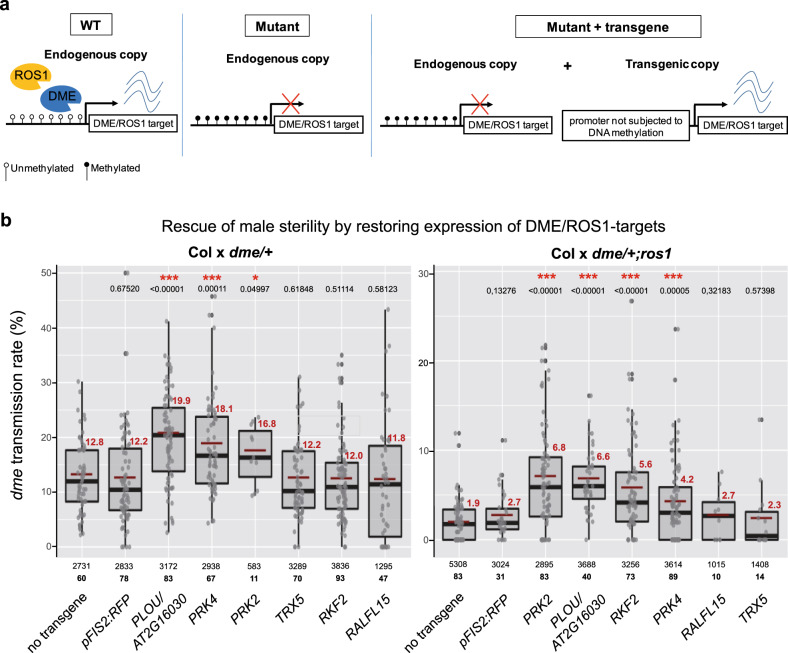
DME/ROS1 target genes are required for male fertility. **a** Strategy to functionally validate the role of DME/ROS1 target genes for pollen tube function. In the wild-type DME/ROS1 target genes become demethylated in VCs, a prerequisite for proper gene activation. In *dme* mutant VCs the demethylation is absent and hence prevents gene expression of the underlying gene. By introducing DME/ROS1 target genes under the control of a DNA methylation-independent, pollen-specific promoter, the gene activity is restored in *dme* VCs and should, at least partially, rescue the male *dme* transmission defect. **b** Box plot showing male *dme* mutant allele transmission frequency of crosses of wild-type (Col-0) mothers with *dme/+* (left graph) and *dme/+;ros1* (right graph) fathers, carrying transgenes to express DME/ROS1 targets independently of DNA methylation in pollen. Transgenes are indicated below the X-axis. *pFIS2:RFP* is a negative control, containing a *FIS2* promoter expressing a reporter gene in the same vector backbone as the other constructs. Individual data points are shown as gray dots and boxes contain 50% of all values, with the second and third quartile separated by the median level; red lines indicate the mean level with corresponding number given in red. The total number of progeny, as well as the replicates (in bold) is given below the graph, representing single siliques in the case of *dme/+* or 1–4 siliques in the case of *dme/+;ros1* for each cross. Pairwise comparison with the *dme*-only crosses revealed significantly elevated *dme* transmission in the case of *AT2G16030/PLOU*, *PRK4*, and *PRK2* in the *dme/+* and *dme/+;ros1* mutant background and *RKF2* in the *dme/+;ros1* background (red stars indicate statistical significance in pairwise comparison with control crosses without transgene: ***p < 0.001; *p < 0.05; the exact *p*-value derived from two-sided *T*-test is shown above the respective cross). Source data underlying Fig. 4b are provided as a Source Data file.

To determine experimentally that repression of these six genes in mutant VCs contributes to paternal sterility, we performed functional complementation assays using transgenes containing a pollen-specific promoter that is independent of DNA methylation changes (see Fig. 4a for a schematic description). Two promoters of different strength were chosen so as to adjust as much as possible transgene expression levels in mutant pollen with those of the native genes in wild-type pollen (Supplementary Data 10). Wild-type and *ros1* plants were transformed and for each construct two independent transgenic progeny (T1) were crossed with *dme/+* or *dme/+;ros1* to obtain single and double mutant T2 progeny, which was then crossed with wild-type mothers to measure paternal transmission of *dme* in the presence of the transgene (Fig. 4b). The re-establishment of gene expression in *dme/+* mutant pollen was verified by RT-qPCR (Supplementary Table 1). Control crosses without the transgene lead, as expected, to low *dme* transmission (12.8% in case of the *dme/+* single mutant and 1.9% in case of the *dme/+;ros1* double mutant; Fig. 4b). Low *dme* transmission was also observed when the *pFIS2-mCherry* transgenic line carrying the same vector backbone as for the DME/ROS1 target transgenes was used as control (12.2% for *dme/+* and 2.7% for *dme/+;ros1*; Fig. 4b). In contrast, re-establishing expression of *PRK2*, *PRK4*, or *AT2G16030* was sufficient to increase significantly *dme* transmission in the single and double mutant backgrounds (Fig. 4b). In addition, although forced pollen expression of *RKF2* had no detectable effect in *dme/+*, it lead to a notable fertility increase in *dme/+;ros1*, similar to that observed with the other three genes (Fig. 4b).

*PRK2* and *PRK4* encode pollen-specific receptor-like kinases (PRKs). Whereas PRK2/AT2G07040 regulates the ROP1 signaling pathway for polarized cell expansion^27^, PRK4/AT3G20190 is thought to mediate the extracellular signals organizing cytoskeleton orientation during polar pollen tube growth^28^. As for *RKF2* (*AT1G19090*), it encodes a putative receptor-like serine threonine kinase that interacts with CATION/H^+^ EXCHANGER10, which has been implied in targeting of pollen tubes to ovules^30,34^. Finally, *AT2G16030* encodes a putative pollen-specific *S*-adenosyl-MTase^33,35^, which we have named *PLOUTOS* (*PLOU*, see “Discussion”). Remarkably, these four genes show strong pollen-specific co-expression (Supplementary Fig. 5a) and, based on publicly available membrane-bound protein-interactome surveys^36,37^, they also define a protein interaction network (Supplementary Fig. 5b).

## Discussion

Our results reveal that the activity of the DNA glycosylase ROS1 is not confined to somatic tissues, and that it contributes to the demethylation of the genome in the VC. Moreover, they demonstrate that DME/ROS1-mediated DNA demethylation plays an essential role in the regulation of genes required for pollen tube function, thus providing a molecular explanation for the male sterility phenotypes observed in *dme/+* and *dme/+;ros1* mutants.

It has been reported that the pollen sterility in *dme/+* mutants is more pronounced in the accession Col-0 than in L*er*^12,14^. Furthermore, ROS1-dependent DNA demethylation varies between accessions in vegetative tissues^38^ and DNA methylation levels in VCs are higher in Col-0 compared to L*er*^39^. This higher DNA methylation level affects mainly the CHH context, consistent with RdDM being especially aggressive in male sex cells, where it targets numerous genes^25^. Thus, we can expect demethylase activity to be more critical in Col-0 than in L*er* to counterbalance the potential silencing of genes required for pollen function. Moreover, genes with strain-independent dense, TE-like DNA methylation tend to be silenced during vegetative growth and expressed specifically in pollen and seeds, presumably as a result of active DNA demethylation in the respective gametophytes^22,40^. Remarkably, three of the genes that are required for male fertility (*AT2G16030*/*PLOU*, *PRK2*, and *RKF2*) show dense TE-like DNA methylation outside the VC (Fig. 3b and Supplementary Fig. 4) and are specifically activated in pollen (Supplementary Fig. 5a). Such heterochomatization of reproductive genes might contribute to their rapid evolution^41^, as cytosines show higher mutation rates when methylated^42^. Be that as it may, whether the demethylation of TEs during reproduction is a side effect of a gene regulatory role or vice versa remains to be determined.

Among the four pollen-specific genes regulated by DME and ROS1, *AT2G16030* was previously uncharacterized. Nonetheless, its protein product is thought to interact with MALE DISCOVERER 2, a receptor-like kinase involved in pollen tube guidance^32,33^. Given that *dme* mutant pollen tubes appear to be blind to maternal signals that direct pollen tube progression, we propose to name gene *AT2G16030* as *PLOUTOS (PLOU)*, after the son of Demeter and god of wealth, who was blinded by Zeus in Greek mythology^43^. Pollen tube progression is governed by multiple molecular interactions with female tissues, through preovular (sporophytic) and ovular (gametophytic) signaling^44–46^ and it has been hypothesized that DNA demethylation is required for the ovule-specific expression of genes encoding cysteine-rich peptides, which could act as pollen tube attractants^40^. Thus, it is tempting to speculate that active DNA demethylation has been co-opted in the male and female reproductive organs to enable the expression of genes underpinning parental communication and reproductive success.

## Methods

### Plant material and growth conditions

Mutant alleles *dme-6* (GK-252E03-014577) and *ros1-3* (WiscDsLox469C11) used in this study are in the Col-0 background and have been characterized previously^18,47^. Seeds where surface-sterilized and sowed on 1/2 Murashige and Skoog (MS) medium plates containing sulfadiazine in case of *dme-6/+* mutant lines or plates without antibiotic selection under sterile conditions. After few days of 4 °C stratification the plates where transferred to 22.5 °C/16 h light–17 °C/8 h dark growth conditions for 10–15 days before transfer to soil under the same growth conditions with 75% humidity control.

### Quantification of *dme* transmission rate

Pre-emasculated Col-0 wild-type flowers were pollinated with pollen from either *dme/+*, *ros1/+*, or *dme/+;ros1* mutants. As the T-DNA insertion responsible for the *dme* mutation confers resistance to sulfadiazine, mature seeds derived from these crosses were sterilized and sowed on 1/2 MS medium containing sulfadiazine, for selection of the *dme* allele-containing plants. To verify proper pollination, siliques were harvested separately and only crosses with at least 20 seeds/silique were taken into account. In general, the total number of seeds per silique did not differ in crosses with Col-0, *ros1*, *dme/+*, and dme/+;*ros1* fathers. We corroborated sulfadiazine resistance with the presence of the T-DNA insertion in the *DME* locus by comparing the numbers of antibiotic resistant plants with those identified as *dme/+* lines by PCR genotyping of two separate populations of the progeny of the same cross (Col_x_*dme/+*): Sulfadizine-resistant: 17.5% (*n* = 480); PCR genotyping: 17.8% (*n* = 185); *p* = 0.9115, Fisher’s exact test. The *ros1* mutant allele segregation was measured by PCR genotyping (see Supplementary Data 11 for primer information). In the mutant vs. wild-type pollen competition assays, limited amount of pollen derived from *dme/+* and *dme/+;ros1* mutants was applied to the stigmata of the pistils. In both cases, the anthers contain approximately half *DME* wild-type and half *dme* mutant pollen (see Supplementary Fig. 1a, b). To verify that fertilization was indeed restricted to few pollen grains, only siliques with less than 25 seeds were taken into account, which corresponds to about half of the normal seed set in Col-0. Mutant allele transmission frequencies were established using Microsoft Excel software version 2013.

### In vivo pollen tube visualization

Pistils from 24 h fertilized flowers were fixed for 2 h in acetic acid/EtOH (1:3) solution, under vacuum at room temperature after which pistils were re-hydrated with successive incubation with 70% EtOH, 50% EtOH, 30% EtOH, and finally distilled water. Pistils were then transferred to 8 M NaOH solution overnight and washed three times with distilled water. Staining was performed by incubating pollinated pistils in Methyl-Blue solution (0.2 mg/ml methyl blue in 100 mM KH_2_PO_4_ pH 11), 2 h, under dark condition at room temperature. After transfer on microscopic slide in 50% glycerol, fluorescence was observed under UV light. Microscopy acquisition were performed on a *Zeiss* Axioskop 2 plus microscope and images were treated using ImageJ v1.52.

### Pollen viability assay

Alexander’s staining were performed to assess pollen viability. Stamens containing mature pollen were treated with few microliter of Alexander solution and observed under light microscope.

### Pollen isolation and RT-qPCR analysis

We used a published protocol (BioProtocols site: *Bio-101*: e67. DOI: 10.21769/BioProtoc.67) with minor changes^48^. Mature flower of about 20 plants were collected and put into 150 mL of cold mannitol (0.3 M) for quadruplicates of Col-0, *ros1*, *dme/+*, and *dme/+;ros1* each. In the case of the transgene complementation lines, duplicates were used. After 2 min of agitation by thorough shaking, the solution was filtered using a 100 µm nylon mesh. Isolated pollen was collected after 5 min centrifugation (3500 × *g* at 4 °C), the pellet rinsed with water and centrifuged again at 10,000 × *g* for 5 min. Purified pollen was stored at −80 °C and the material homogenized with metallic beads using a Qiagen tissue-lyser (2 × 1′ 30 Hz) to perform total RNA extraction using a Qiagen RNeasy Mini Kit, following the manufacturer’s instruction. RNA integrity was verified on gel after speZZctrometric quantification and cDNA synthesis was carried out with 10 μg of RNA for Col-0 wild type and mutants and 1 μg of RNA in the case of the transgene complementation lines using the Invitrogen Superscript IV Reverse Transcriptase, following the manufacturer’s instructions. qPCR was done employing Roche SYBR Green reagent and a Roche LightCycler 480 with technical duplicates for each sample and primers (Supplementary Data 11) were tested using the Roche LightCycler Software 4.1 for specificity and >90% efficiency beforehand.

### Cell sorting of VCs and SCs

Pollen nuclei of wild-type (Col-0) and mutant *dme/+*, *ros1*, and *dme/+;ros1* plants were purified by FACS using SYBR Green staining as previously described^49,50^. Briefly, open flowers were collected into a 50 mL falcon tubes and vortexed in 10 mL of Galbraith buffer (45 mM MgCl_2_, 30 mM Sodium Citrate, 20 mM MOPS, 1% Triton-100 pH 7.0) for 3 min, at room temperature. This crude fraction was then filtered through Miracloth (Calbiochem) and centrifuged for 1 min at 2600 × *g* to concentrate the pollen fraction. The pollen was then transferred to a 1.5 mL Eppendorf tube containing ~100 μL of acid-washed glass beads (425–600 μm, Sigma) and vortexed continuously at maximum speed for 3 min, to break the pollen cell wall. The fraction containing the released nuclei was then filtered through a 10 μm mesh (Celltrics, Sysmex-Partec) to exclude pollen debris and stained with SYBR Green dye (Lonza). FACS was performed using a FACSAria IIU cell sorter (BD Biosciences), using the integrated FACSDiva v6.1 software with 70 µ nozzle at 70 psi. A 488 nm laser was used for SYBR Green excitation, which was detected by a 530/30 nm band-pass filter (Supplementary Fig. 2). Approximately 500,000 nuclei from each genotype and cell type were purified, and genomic DNA was extracted using the Masterpure kit (Epicenter).

### Whole-genome bisulfite sequencing and DMR calling

Genomic DNA was fragmented by Covaris to ~300 bp and fragments were end-repaired, A-tailed, and ligated to methylated Illumina adaptors (Bioo Scientific). Ligated fragments were bisulfite treated with the EZ DNA Methylation-Gold Kit (Zymo) and PCR-enriched with *KAPA* high-fidelity Uracil+ polymerase (Roche). Reads were preprocessed with Trimmomatic v0.32^51^ to remove adaptors and Bismark v0.16.3^52^ was used to map filtered reads to the *Arabidopsis* TAIR10 genome reference, to calculate methylation at each individual cytosine. DMRs were defined as 50 bp bins containing at least 2, 3, or 4 differentially methylated mCGs, mCHGs or mCHHs with an absolute methylation difference of 0.40, 0.25, and 0.10, respectively. Bins localizing within 400 bp of each other were merged. A summary of all genome-wide bisulfite sequencing data generated in this study is presented in Supplementary Data 1 and is accessible through the NCBI’s Gene Expression Omnibus (GSE141154).

### GO-term analysis

The DAVID gene functional classification tool^53^ was used to identify categories of biological function of genes associated to hypo-CG-DMRs detected in the comparison between wild-type VCs vs. SCs, as well as wild-type vs. *dme/+* and *dme/+;ros1* VCs. TAIR10 genome was used as background. We used the standard parameters to detect over-represented GO terms for biological function, giving Fisher’s exact *p*-values, fold-enrichment scores, and number of genes from our gene-list found in a given term.

### Plasmid construction and plant transformation

Functional complementation assay was performed by using the Multisite Gateway Technology (Invitrogen). Promoters were PCR-amplified from genomic DNA and inserted into the *pENTR5’-TOPO* vector using the pENTR5′-TOPO TA Cloning Kit (Invitrogen). CDSs of candidate genes were amplified by PCR on cDNA extracted from mature pollen, using forward and reverse primers containing attB1 and attB2 sites, respectively (Supplementary Data 11). Gateway BP reaction was used to insert amplified CDS into the *pDONR207* vector. We used Gateway LR reaction to clone the *ANX2* (*AT5G28680*) promoter region in combination with the CDS of *PRK2*, *PRK4*, and *RALFL15*, as well as the regulatory sequence of *Raba4D* (AT3G12160) in front of *RKF2*, *TRX5*, and *PLOU* (*AT2G16030*) into the *pDestination* vector *pB7m24GW3*. Transformation of *Agrobacterium tumefaciens* (strain GV3101) were then performed by incubation with 1 µg of the produced vector (5 min on ice), followed by 5 min freezing in liquid nitrogen. After 1 min recovery at 37 °C, 1 mL of Luria-Bertani (LB) medium were added for a 2 h incubation at 28 °C (with shaking), plated on LB containing gentamycin, rifampicin + spectinomycin, and incubated 48 h at 28 °C. Plants were transformed by dipping in *A. tumefaciens* solution. Primers used to generate constructs are listed in Supplementary Data 11.

### Reporting summary

Further information on research design is available in the Nature Research Reporting Summary linked to this article.

## Supplementary information

Supplementary Information

Peer Review File

Reporting Summary

Description of Additional Supplementary Files

Supplementary Dataset 1

Supplementary Dataset 2

Supplementary Dataset 3

Supplementary Dataset 4

Supplementary Dataset 5

Supplementary Dataset 6

Supplementary Dataset 7

Supplementary Dataset 8

Supplementary Dataset 9

Supplementary Dataset 10

Supplementary Dataset 11

## Data Availability

Data supporting the findings of this work are available within the paper and its Supplementary Information files. A reporting summary for this article is available as a Supplementary Information file. The datasets and plant materials generated and analyzed during the current study are available from the corresponding author (D.B.) upon request. WGBS data that support the findings of this study have been deposited at NCBI’s Gene Expression Omnibus under accession number GSE141154. Source Data are provided with this paper.

## Source data

Source Data
