# Supplementary material for: Male fertility in Arabidopsis requires active DNA demethylation of genes that control pollen tube function
Source: Nat Commun. 2021 Jan 18;12:410. doi: 10.1038/s41467-020-20606-1 (PMC7813888; doi:10.1038/s41467-020-20606-1)
Supplement: Supplementary file 1 — Supplementary Information [file 41467_2020_20606_MOESM1_ESM.pdf]

Male fertility in Arabidopsis requires active DNA demethylation of genes that control pollen tube function

Khouider *et al.*

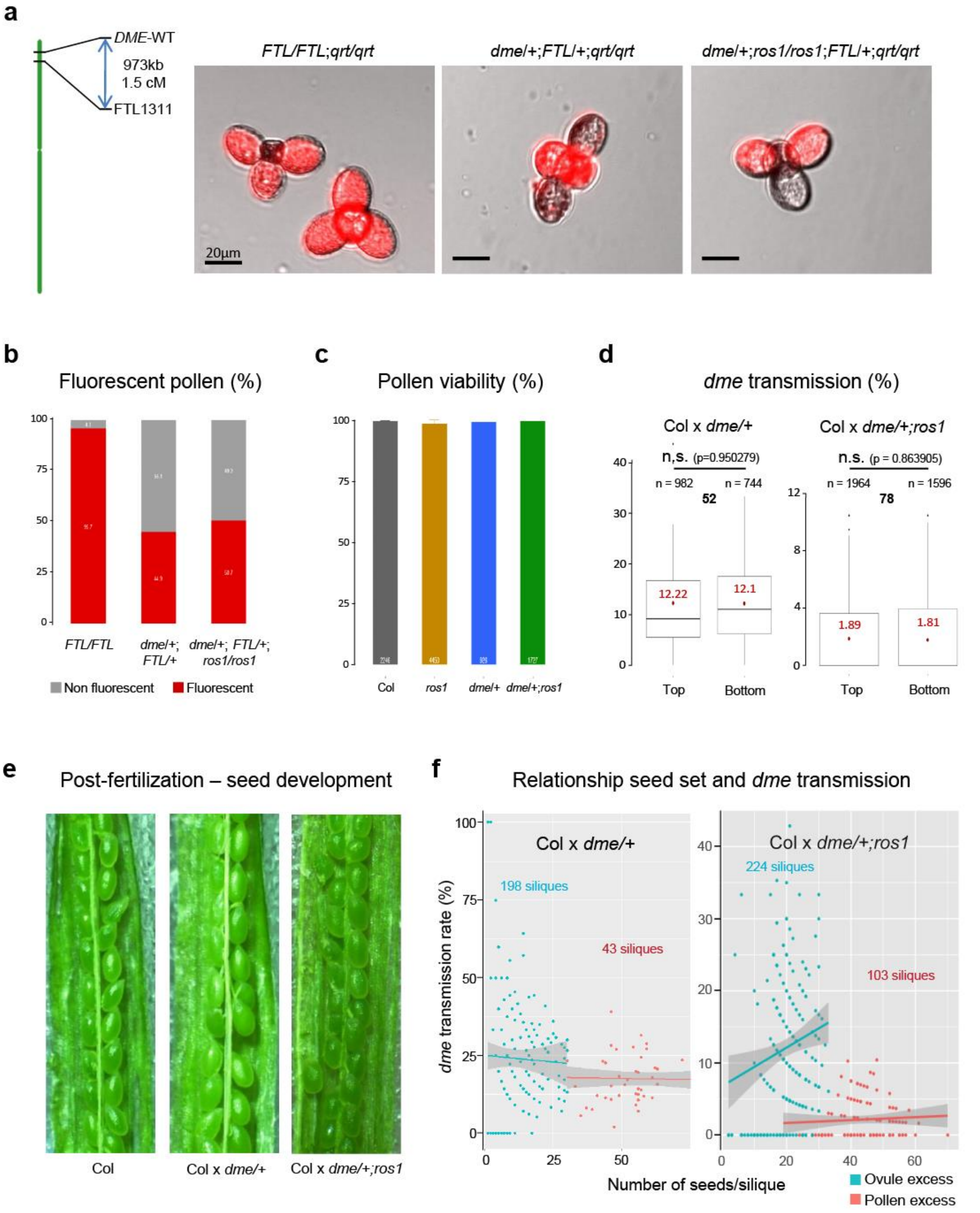

Supplementary Fig. 1. DME/ROS1-activity in pollen is specifically required for pollen tube progression. (a) Schematic representation of the *DME* locus and FTL1311 fluorescent marker [1] position on chromosome 5 (left) and fluorescent images of F2 lines segregating after crosses with *dme/+* or *dme/+;ros1*. Representative images show pollen tetrads instead of free floating pollen grains due to the *qrt* mutant background, leaving the haploid tetrad after meiosis attached together [1] and have been observed independently for at least three times/sample. (b) Quantification of fluorescent/non-fluorescent pollen grains in the progeny of the parental FTL1311 line and the *dme* and *dme ros1*-containing F2s. All

F2 selected for *dme* showed fluorescence in approximately half the pollen grains, indicating physical coupling and absence of meiotic distortion. (c) Percentage of positively stained pollen grains after Alexander staining, used to measure pollen viability; number of counted pollen indicated below the bars. (d) Comparison of *dme* mutant allele transmission frequency in seeds in the top or the bottom half of *dme/*+ and *dme/*+*;ros1*-pollinated siliques. Boxes represent 50% of all values with 2nd and 3rd quartile separated by the median value indicated as black line. Red rhomb indicates the mean value, given in %. The number of individuals in the progeny is given above the bars together with the number of siliques in bold and pairwise comparison did not reveal significant differences (Fisher exact test). (e) Representative examples of siliques containing seeds after crosses with either Col-0 wild type, *dme/*+ or *dme/*+*;ros1*, observed at least five times independently. (f) Correlation of seed set and *dme* transmission rate in crosses with either pollen excess (red dots) or ovule excess (blue dots) in crosses of Col-0 wild-type mothers with either *dme/*+ (left) or *dme/*+*;ros1* (right) fathers. Number of siliques is given and with each data point representing seed set (number of seeds per silique) on the X-axis and mutant transmission rate (% *dme*) on the Y-axis. The linear regression line correlates seed-set and *dme* transmission rate within a 95% confidence interval (grey error band). In case of lesser competition between mutant and wild-type pollen in crosses with fewer pollen one might expect a negative correlation between seed-set and *dme* transmission rate, which is not the case. Source data underlying Supplementary Figure 1b, 1d, and 1f are provided as a Source Data file.

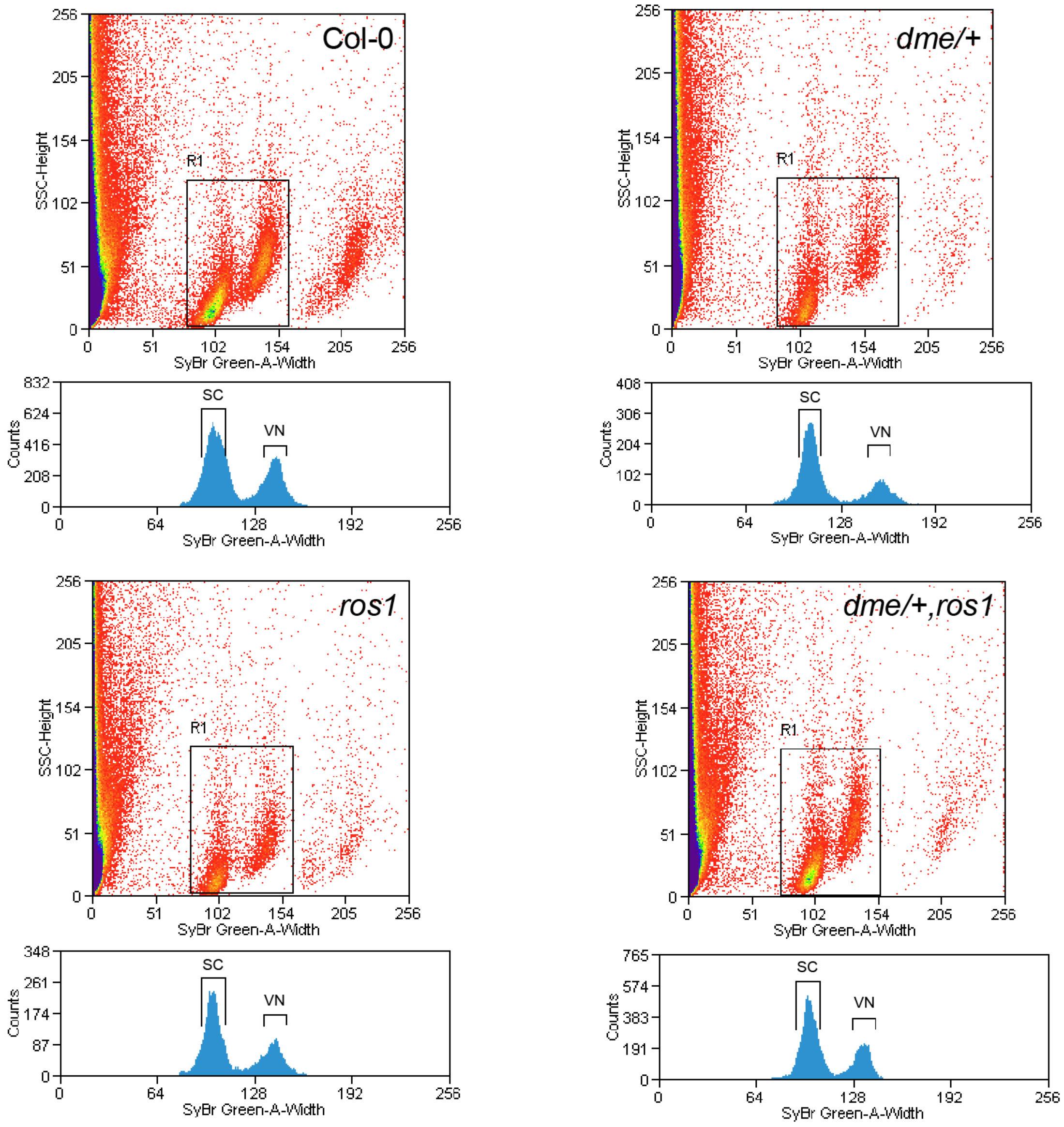

Supplementary Fig. 2. FACS profiles to isolate VC and SC of wild type and mutant pollen. SSC vs FSC density plots (upper graphs) as well as the cell number/count profile (lower graphs) are shown for each sample. Gate (R1) has been applied to remove debris. VN = vegetative nucleus, SC = sperm cell.

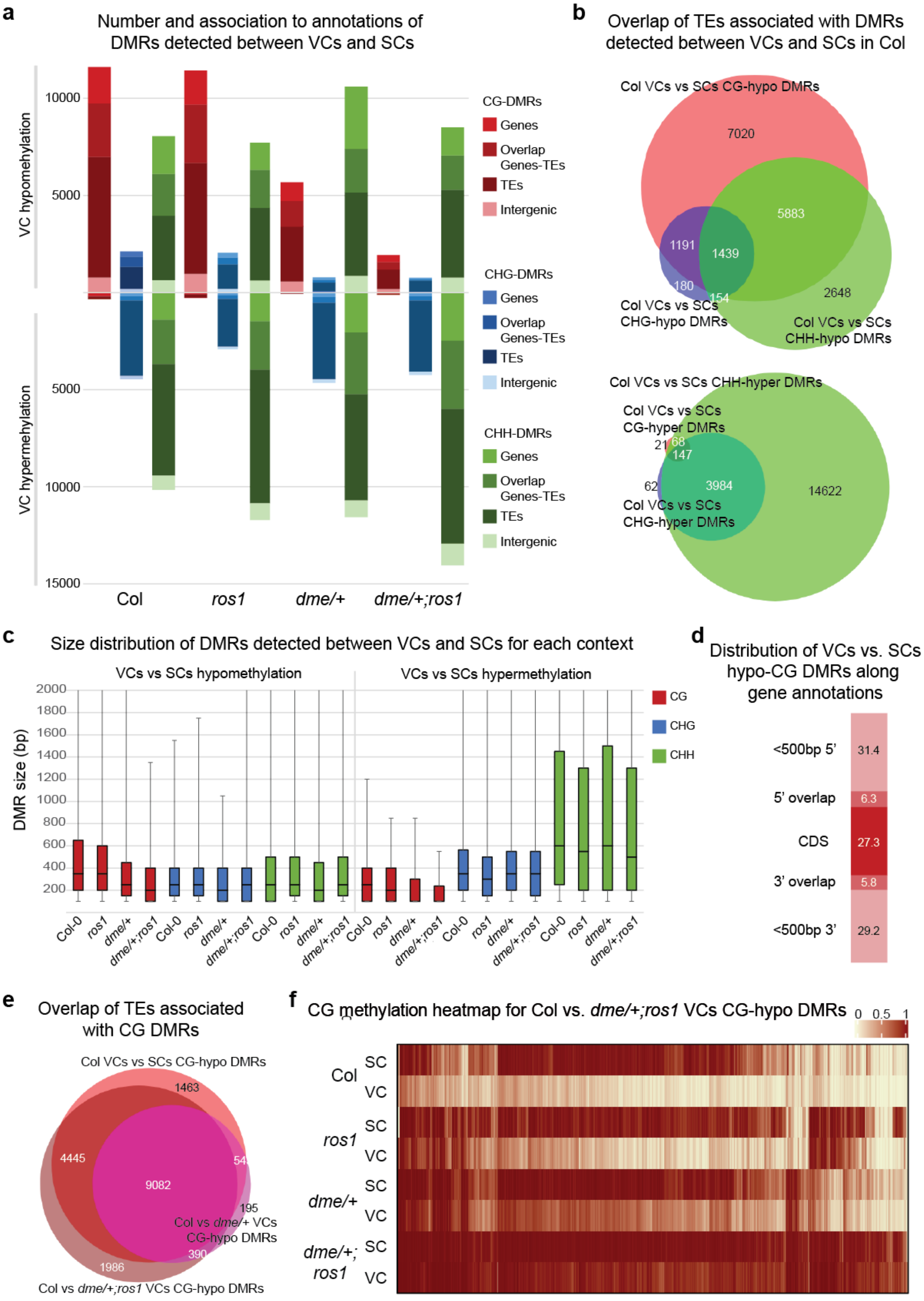
a
Number and association to annotations of DMRs detected between VCs and SCs
VC hypomethylation
VC hypermethylation
10000
5000
5000
10000
15000
Col
ros1
dme/+
dme/+;ros1
CG-DMRs
Genes
Overlap Genes-TEs
TEs
Intergenic
CHG-DMRs
Genes
Overlap Genes-TEs
TEs
Intergenic
CHH-DMRs
Genes
Overlap Genes-TEs
TEs
Intergenic
b
Overlap of TEs associated with DMRs detected between VCs and SCs in Col
Col VCs vs SCs CG-hypo DMRs
7020
5883
1191
1439
180
154
2648
Col VCs vs SCs CHG-hypo DMRs
Col VCs vs SCs CHH-hypo DMRs
Col VCs vs SCs CHH-hyper DMRs
Col VCs vs SCs CG-hyper DMRs
21
68
147
62
3984
14622
Col VCs vs SCs CHG-hyper DMRs
c
Size distribution of DMRs detected between VCs and SCs for each context
VCs vs SCs hypomethylation
VCs vs SCs hypermethylation
DMR size (bp)
2000
1800
1600
1400
1200
1000
800
600
400
200
CG
CHG
CHH
Col-0
ros1
dme/+
dme/+;ros1
d
Distribution of VCs vs. SCs hypo-CG DMRs along gene annotations
<500bp 5'
31.4
5' overlap
6.3
CDS
27.3
3' overlap
5.8
<500bp 3'
29.2
e
Overlap of TEs associated with CG DMRs
Col VCs vs SCs CG-hypo DMRs
1463
4445
9082
54
195
Col vs dme/+ VCs CG-hypo DMRs
390
1986
Col vs dme/+;ros1 VCs CG-hypo DMRs
f
CG methylation heatmap for Col vs. dme/+;ros1 VCs CG-hypo DMRs
0
0.5
1
Col
SC
VC
ros1
SC
VC
dme/+
SC
VC
dme/+; ros1
SC
VC

Supplementary Fig. 3. DME and ROS1 are required for demethylation of the vegetative cell in pollen. (a) Number and association to annotation units of DMRs detected between vegetative cells (VCs) and sperm cells (SCs) in pollen for each sequence context and each genotype are shown as indicated in the graph. (b) Venn diagram displaying the number and overlap of TE annotations associated with hypomethylation (above) or hypermethylation (below) in VCs compared to SCs in Col-0 wild type for each context as indicated in the graph. (c) Box-plot of DMRs as in (a) with respect to size distribution with the boxes representing 50% of all DMRs and the horizontal line indicating the median value separating the 2nd and 3rd quartile. (d) Distribution of CG-hypo DMRs detected between VCs and SCs in Col-0 wild type relative to the position of the transcription unit of DMR-associated protein coding genes as indicated in the graph. (e) Venn diagram showing the number and overlap of TE annotations associated with hypomethylation in VCs versus SCs in Col-0 wild type, as well as in wild-type versus mutant (*dme/+* and *dme/+;ros1*) VCs as indicated in the graph. (f) Heatmap illustrating the absolute CG methylation level for CG-hypo DMRs detected between wild-type (Col-0) and *dme/+;ros1* VCs (n = 14960) for SCs and VCs for each genotype indicated for each line. DMRs in the columns were sorted by complete linkage hierarchical clustering using Euclidean distance.

Genes involved in pollen tube function with ectopic DNA methylation in mutant VCs

Class I : Demethylation of pollen-specific DNA methylation

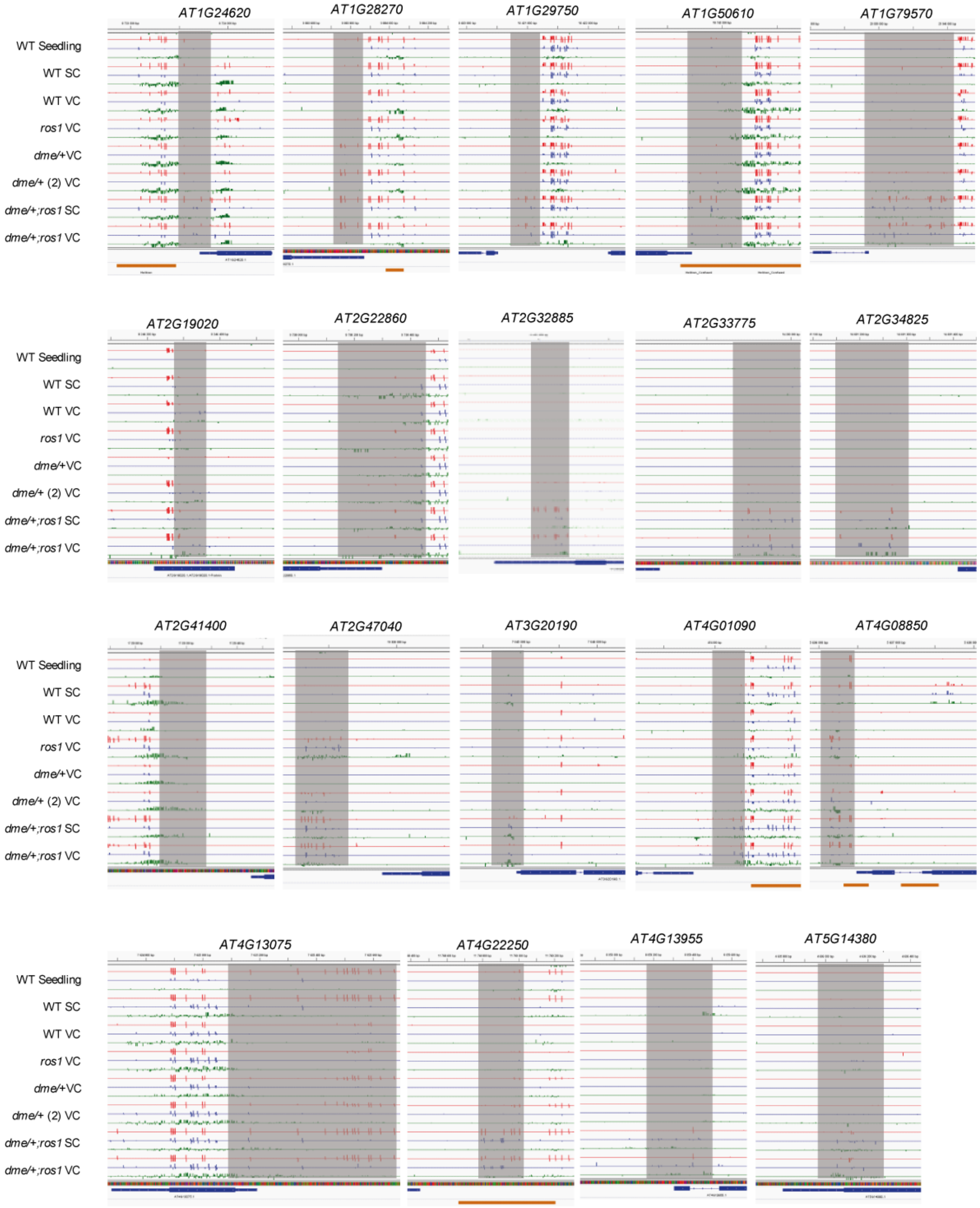

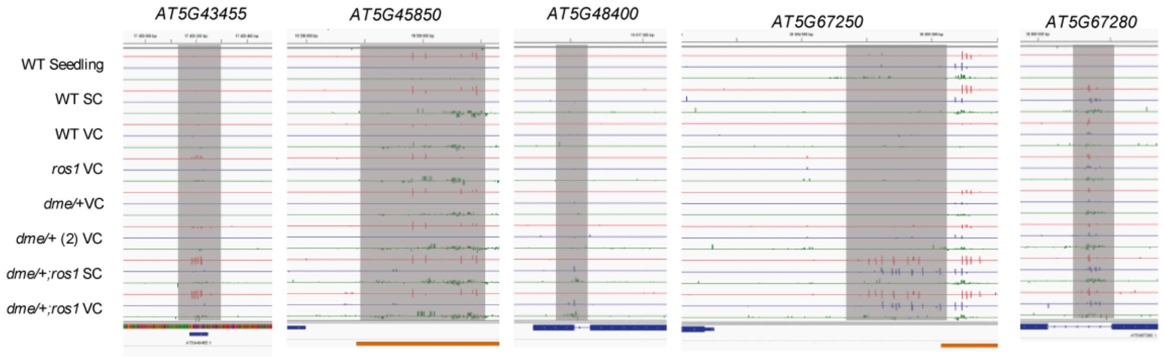

Class II : VC-specific DNA demethylation over commonly methylated region

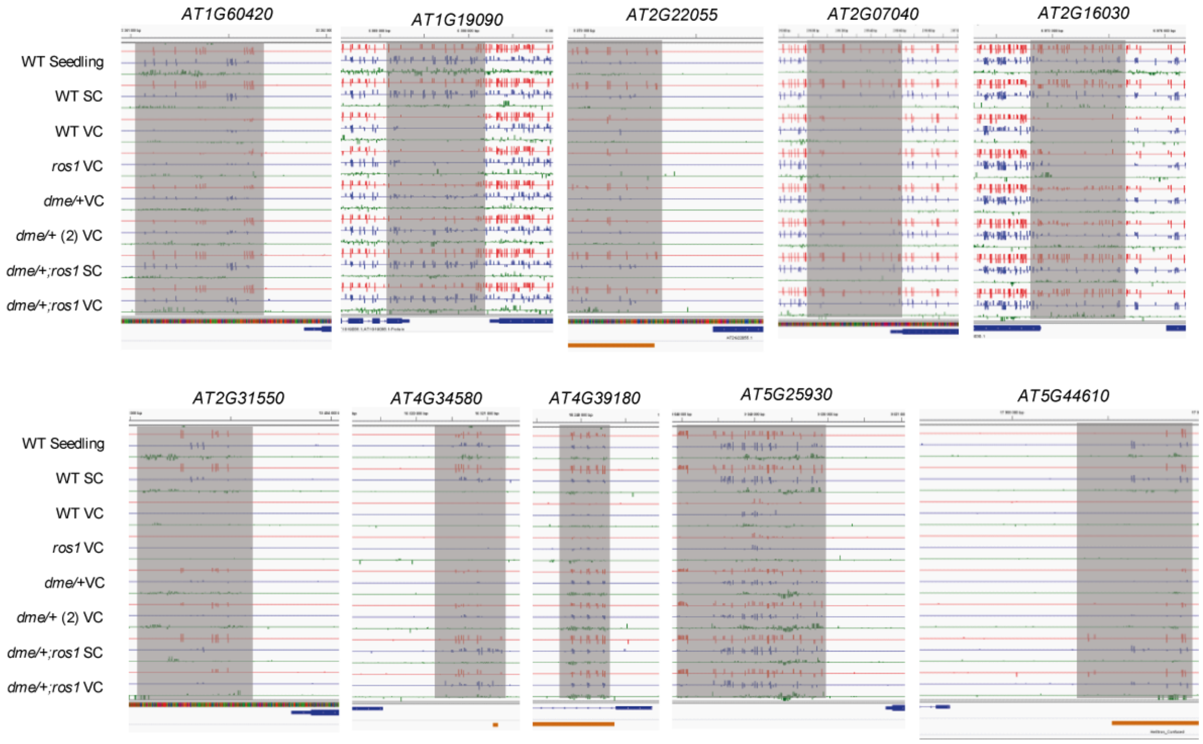

Supplementary Fig. 4. DNA demethylation in the vegetative cell in the upstream region of genes implicated in pollen tube function. Genome-browser views showing absolute DNA methylation levels for all three contexts (as in Fig. 3a) of genes involved in pollen tube function (see Supplementary Data 9) with elevated DNA methylation in *dme/+;ros1* compared to wild-type VC. Beside the VCs samples the DNA methylation of wild-type seedlings and SC as well as the SC of *dme/+;ros1* and the VC of published *dme/+* mutant VC is shown [dme/+ (2)[2]].

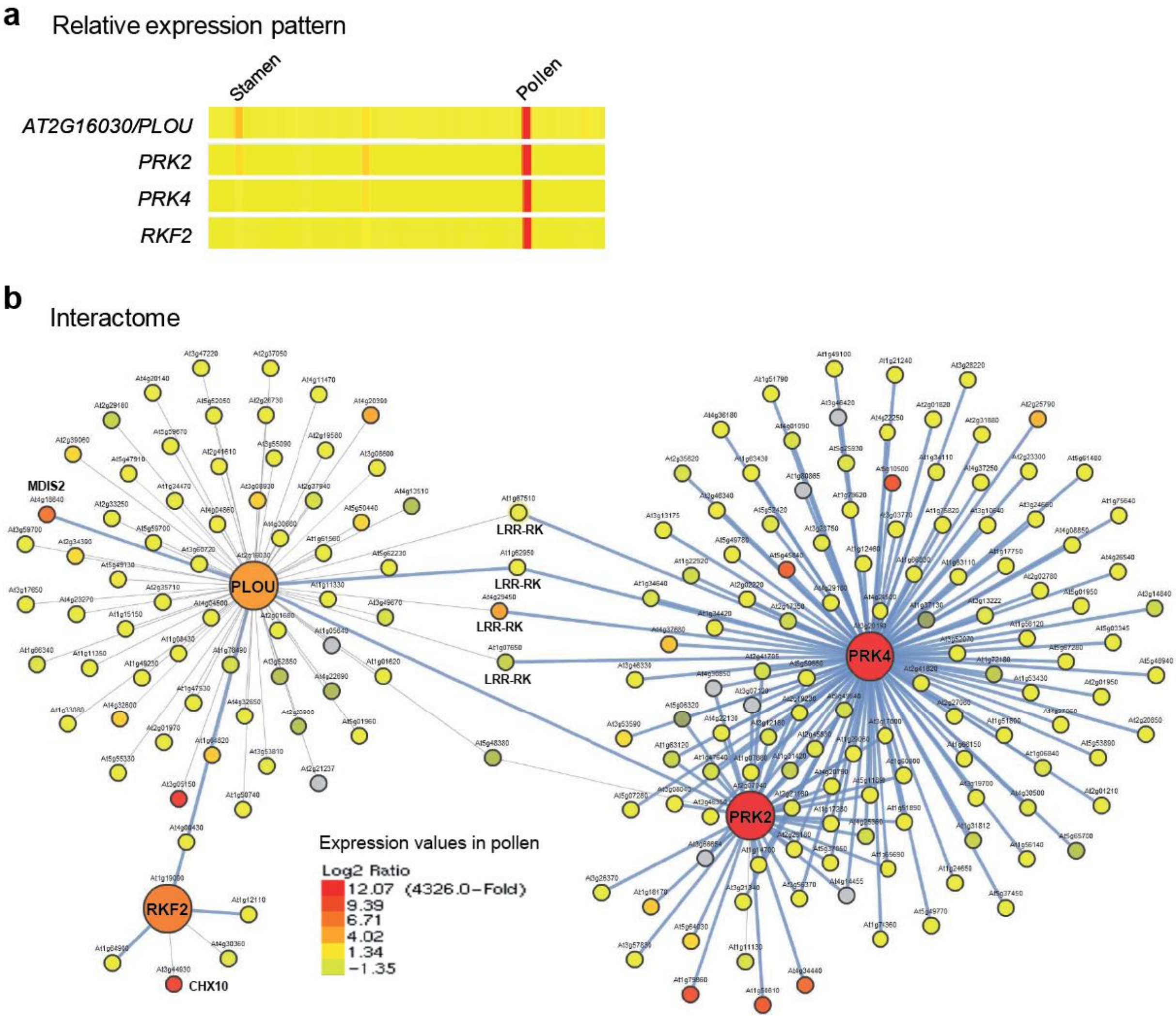

Supplementary Fig. 5. Interaction network of DME-ROS1 targets. (a) Relative gene expression pattern for four DME/ROS1 target genes implicated in pollen tube function using the Expression Angler online tool (http://bar.utoronto.ca/ExpressionAngler). (b) Protein-protein interaction of four DME/ROS1 target genes (PLOU, PRK2, PRK4 and RKF2; big nodes) with color-coded relative expression level in mature pollen. Only published interactions are shown, based on the implemented data base of the Arabidopsis Interactions Viewer site (http://bar.utoronto.ca/interactions/cgi-bin/arabidopsis_interactions_viewer.cgi). Bold grey node-connecting lines indicate experimentally validated interactions. DME/ROS1 targets (big nodes) are labeled as well as putative membrane-bound signaling components potentially involved in pollen function and/or bridging DME/ROS1 targets with each other.

Supplementary Table 1. Relative gene expression level of cDNAs used in the transgenic complementation lines

Line	*AT2G39805*	*AT3G58480*	*AT5G28680*	*AT3G12160*	*PLOU*	Transgene/WT*
Col (wild type)	1 ± 0.07	1 ± 0.11	1 ± 0.14	1 ± 0.11	1 ± 0.06	-
dme/+	1.03 ± 0.03	1.06 ± 0.03	0.93 ± 0.02	0.98 ± 0.03	0.56 ± 0.27	-
PLOU_#1 (*dme*/+)	0.80 ± 0.01	1.03 ± 0.09	1.17 ± 0.08	1.02 ± 0.03	*4.27 ± 0.04**	4.27 ± 0.04
PLOU_#2 (*dme*/+)	0.99 ± 0.07	1.05 ± 0.04	1.05 ± 0.05	0.91 ± 0.02	*2.01 ± 0.14**	2.01 ± 0.14
PRK2_#1 (*dme*/+)	1.04 ± 0.03	1.05 ± 0.02	0.94 ± 0.05	0.96 ± 0.04	0.73 ± 0.48	2.49 ± 0.01
PRK2_#2 (*dme*/+)	0.94 ± 0.01	0.99 ± 0.01	0.98 ± 0.02	1.08 ± 0.02	0.73 ± 0.10	1.87 ± 0.03
PRK4_#1 (*dme*/+)	1.11 ± 0.13	1.11 ± 0.04	0.84 ± 0.24	0.98 ± 0.15	0.55 ± 0.06	1.51 ± 0.41
PRK4_#2 (*dme*/+)	0.84 ± 0.01	1.09 ± 0.03	1.01 ± 0.02	1.08 ± 0.05	0.63 ± 0.09	1.96 ± 0.06
RALFL15_#1 (*dme*/+)	0.96 ± 0.13	0.98 ± 0.08	1.07 ± 0.01	1.00 ± 0.20	0.53 ± 0.11	1.37 ± 0.27
RALFL15_#2 (*dme*/+)	0.94 ± 0.17	0.99 ± 0.01	1.01 ± 0.07	1.07 ± 0.13	0.71 ± 0.05	1.17 ± 0.10
RKF2_#1 (*dme*/+)	0.96 ± 0.03	1.01 ± 0.02	1.06 ± 0.01	0.95 ± 0.05	0.49 ± 0.06	1.22 ± 0.10
RKF2_#2 (*dme*/+)	0.79 ± 0.04	1.04 ± 0.01	1.10 ± 0.10	1.09 ± 0.06	0.73 ± 0.12	1.16 ± 0.12
TRX5_#1 (*dme*/+)	1.01 ± 0.01	0.98 ± 0.03	1.07 ± 0.02	0.93 ± 0.01	0.54 ± 0.01	1.77 ± 0.04
TRX5_#2 (*dme*/+)	0.95 ± 0.09	0.99 ± 0.02	1.09 ± 0.02	0.97 ± 0.05	0.60 ± 0.08	2.13 ± 0.51

For each genotype the level of 4 reference transcripts, as well as *PLOU* as a control of the *dme/+* mutant background and the respective transgene are shown. All values are given relative to wild type (Col-0), including standard error of duplicates for each line. For all transgenes two independent lines were used.

* Transgenic and endogenous origin of the respective transcripts cannot be discriminated, hence the

Supplementary References

1. Francis, K. E. *et al.* Pollen tetrad-based visual assay for meiotic recombination in Arabidopsis. *Proc. Natl. Acad. Sci. U.S.A.* **104**, 3913–3918 (2007).
2. Ibarra, C. A. *et al.* Active DNA demethylation in plant companion cells reinforces transposon methylation in gametes. *Science* **337**, 1360–1364 (2012).
