# Supplementary material for: Male fertility in Arabidopsis requires active DNA demethylation of genes that control pollen tube function
Source: Nat Commun. 2021 Jan 18;12:410. doi: 10.1038/s41467-020-20606-1 (PMC7813888; doi:10.1038/s41467-020-20606-1)
Supplement: Supplementary file 3 — Reporting Summary [file 41467_2020_20606_MOESM3_ESM.pdf]

Corresponding author(s): Daniel Bouyer
Vincent Colot

Last updated by author(s): Dec 9, 2020

Reporting Summary

Nature Research wishes to improve the reproducibility of the work that we publish. This form provides structure for consistency and transparency in reporting. For further information on Nature Research policies, see our Editorial Policies and the Editorial Policy Checklist.

Statistics

For all statistical analyses, confirm that the following items are present in the figure legend, table legend, main text, or Methods section.

n/a	Confirmed	
☐	☒	The exact sample size (*n*) for each experimental group/condition, given as a discrete number and unit of measurement
☐	☒	A statement on whether measurements were taken from distinct samples or whether the same sample was measured repeatedly
☐	☒	The statistical test(s) used AND whether they are one- or two-sided<br>*Only common tests should be described solely by name; describe more complex techniques in the Methods section.*
☒	☐	A description of all covariates tested
☒	☐	A description of any assumptions or corrections, such as tests of normality and adjustment for multiple comparisons
☐	☒	A full description of the statistical parameters including central tendency (e.g. means) or other basic estimates (e.g. regression coefficient) AND variation (e.g. standard deviation) or associated estimates of uncertainty (e.g. confidence intervals)
☐	☒	For null hypothesis testing, the test statistic (e.g. *F*, *t*, *r*) with confidence intervals, effect sizes, degrees of freedom and *P* value noted<br>*Give P values as exact values whenever suitable.*
☒	☐	For Bayesian analysis, information on the choice of priors and Markov chain Monte Carlo settings
☒	☐	For hierarchical and complex designs, identification of the appropriate level for tests and full reporting of outcomes
☒	☐	Estimates of effect sizes (e.g. Cohen's *d*, Pearson's *r*), indicating how they were calculated

Our web collection on statistics for biologists contains articles on many of the points above.

Software and code

Policy information about availability of computer code

Data collection	Microsoft Excel version 2013 was used for establishing mutant allele transmission rates, RT-qPCR calculations, and visualization of methylated regions. Roche Lightcycler software 4.1 was used to establish primer efficiencies and qPCR Cp values. BD Lifescience FACSDiva v6.1 software was used to isolate vegetative- and sperm cell samples from wild-type and mutant pollen. ImageJ v1.52 was used to process microscope images. Adobe Illustrator v15.0 was used to generate figure files.
Data analysis	Trimmomatic v0.32 was used to pre-process Illumina reads. Bismark v0.16.3 was used to map bisulfite converted DNA sequencing reads to TAIR 10 reference genome.

For manuscripts utilizing custom algorithms or software that are central to the research but not yet described in published literature, software must be made available to editors and reviewers. We strongly encourage code deposition in a community repository (e.g. GitHub). See the Nature Research guidelines for submitting code & software for further information.

Data

Policy information about availability of data

All manuscripts must include a data availability statement. This statement should provide the following information, where applicable:

- Accession codes, unique identifiers, or web links for publicly available datasets
- A list of figures that have associated raw data
- A description of any restrictions on data availability

WGBS data that support the findings of this study have been deposited at NCBI's Gene Expression Omnibus under accession number GSE141154: https://www.ncbi.nlm.nih.gov/geo/query/acc.cgi?acc=GSE141154. Data supporting the findings of this work are available within the paper and its Supplementary Information files. Following figures have associated raw data source files:
Fig. 1a, c, 3b, 4c

Suppl. Fig. 1b, d, f

Field-specific reporting

Please select the one below that is the best fit for your research. If you are not sure, read the appropriate sections before making your selection.

- [x] Life sciences
- [ ] Behavioural & social sciences
- [ ] Ecological, evolutionary & environmental sciences

For a reference copy of the document with all sections, see nature.com/documents/nr-reporting-summary-flat.pdf

Life sciences study design

All studies must disclose on these points even when the disclosure is negative.

Sample size	Sample size was calculated based on the statistical requirements to distinguish significant differences in Student T-test for comparisons with elevated event size or Fisher-exact test for comparisons with low number of events.
Data exclusions	no data was excluded
Replication	Mutant allele transmission frequencies were established several times independently of each other, using several biological replicates and using single siliques to verify proper pollination. Four biological were used in RT-qPCR experiments to establish transcript levels for wild type and mutants and duplicates in case of the transgene-expression analysis. Two technical replicates were used in qRT-PCR and comparable levels verified before proceeding.<br>All replication attempts were successful and consistent.
Randomization	We did not randomize sample due to fear of confounding effects. We were careful to perform experiments under conditions as comparable as possible with highly homogenic (plant) material. For instance one set of experiments was always performed completely within a limited time frame to avoid deviations of growth conditions.
Blinding	Measurement of mutant allele transmission in combination with transgenic complementation experiments were blinded. Beside these mentioned experiments, we did not use blinding for group allocations as we worked with high numbers of genetically identical (plant) material allocated to their respective groups.

Reporting for specific materials, systems and methods

We require information from authors about some types of materials, experimental systems and methods used in many studies. Here, indicate whether each material, system or method listed is relevant to your study. If you are not sure if a list item applies to your research, read the appropriate section before selecting a response.

Materials & experimental systems

n/a	Involved in the study	
x		Antibodies
x		Eukaryotic cell lines
x		Palaeontology and archaeology
x		Animals and other organisms
x		Human research participants
x		Clinical data
x		Dual use research of concern

Methods

n/a	Involved in the study	
x		ChIP-seq
	x	Flow cytometry
x		MRI-based neuroimaging

Flow Cytometry

Plots

Confirm that:

- [x] The axis labels state the marker and fluorochrome used (e.g. CD4-FITC).
- [x] The axis scales are clearly visible. Include numbers along axes only for bottom left plot of group (a 'group' is an analysis of identical markers).
- [x] All plots are contour plots with outliers or pseudocolor plots.
- [x] A numerical value for number of cells or percentage (with statistics) is provided.

Methodology

Sample preparation	Pollen nuclei were purified by FACS using SYBR Green staining. Open flowers were collected into a 50 ml falcon tubes, and

vortexed in 10 ml of Galbraith buffer (45 mM MgCl2, 30 mM Sodium Citrate, 20 mM MOPS, 1% Triton-100, pH to 7.0) for 3 minutes, at room temperature. This crude fraction was then filtered through Miracloth (Calbiochem) and centrifuged for 1 minute at 2600 g to concentrate the pollen fraction. The pollen was then transferred to a 1.5 ml eppendorf tube containing approximately 100l of acid-washed glass beads (425–600m, Sigma), and vortexed continuously at maximum speed for 3 minutes in order to break the pollen cell wall. The fraction containing the released nuclei was then filtered through a 10m mesh (Celltrics, Sysmex-Partec) to exclude pollen debris, and stained with SYBR Green dye (Lonza).

Instrument

FACS was performed using a FACSAria IIU cell sorter (BD Biosciences) with 70µ nozzle at 70psi. A 488-nm laser was used for SYBR Green excitation, which was detected by a 530/30 nm band-pass filter

Software

FACSDiva v6.1 software (BD Biosciences)

Cell population abundance

Sperm cells and vegetative nucleus were separated based on size and fluorescence intensity (see Supplemental Figure 2). The purity was checked based on size under fluorescent microscope and is more than 97% for both type of nuclei. Approximately 500,000 from each cell type were isolated.

Gating strategy

A 488-nm laser was used for SYBR Green excitation, which was detected by a 530/30 nm band-pass filter. Gating was based on FSC and pulse width and only those events which present a homogenous SSC profile were used (see Supplemental Figure 2).

☒ Tick this box to confirm that a figure exemplifying the gating strategy is provided in the Supplementary Information.
