# Supplementary material for: Male fertility in Arabidopsis requires active DNA demethylation of genes that control pollen tube function
Source: Nat Commun. 2021 Jan 18;12:410. doi: 10.1038/s41467-020-20606-1 (PMC7813888; doi:10.1038/s41467-020-20606-1)
Supplement: Supplementary file 4 — Description of Additional Supplementary Files [file 41467_2020_20606_MOESM4_ESM.pdf]

Description of Additional Supplementary Files

Supplementary Data 1. WGBS General data. CSV file containing information concerning the whole genome bisulfite sequencing samples generated in this study.

Supplementary Data 2. DMRs VC vs. SC Col. CSV file containing all DMRs detected between SC and VC of wild-type Col-0 pollen for all sequence contexts, indicating its genomic position and overlap with annotation (gene or TE) of up to 500bp 5’ or 3’ of the annotation unit.

Supplementary Data 3. DMRs VC vs. SC ros1. CSV file containing all DMRs in all sequence contexts detected between SC and VC of ros1-3 pollen for all sequence contexts, indicating its genomic position and overlap with annotation (gene or TE) of up to 500bp 5’ or 3’ of the annotation unit.

Supplementary Data 4. DMRs VC vs. SC dme. CSV file containing all DMRs in all sequence contexts detected between SC and VC of dme-6/+ - derived pollen for all sequence contexts, indicating its genomic position and overlap with annotation (gene or TE) of up to 500bp 5’ or 3’ of the annotation unit.

Supplementary Data 5. DMRs VC vs. SC dmeros1. CSV file containing all DMRs in all sequence contexts detected between SC and VC of dme-6/+;ros1 - derived pollen for all sequence contexts, indicating its genomic position and overlap with annotation (gene or TE) of up to 500bp 5’ or 3’ of the annotation unit.

Supplementary Data 6. DMRs VC Col vs. ros1. CSV file containing all DMRs in all sequence contexts detected between VCs of Col-0 wild-type and ros1-3 - derived pollen for all sequence contexts, indicating its genomic position and overlap with annotation (gene or TE) of up to 500bp 5’ or 3’ of the annotation unit.

Supplementary Data 7. DMRs VC Col vs. dme. CSV file containing all DMRs in all sequence contexts

detected between VCs of Col-0 and dme-6/+ - derived pollen for all sequence contexts, indicating its genomic position and overlap with annotation (gene or TE) of up to 500bp 5' or 3' of the annotation unit.

Supplementary Data 8. DMRs VC Col vs. dmeros1. CSV file containing all DMRs in all sequence contexts detected between VC of Col-0 wild-type and dme-6/+;ros1-3 - derived pollen for all sequence contexts, indicating its genomic position and overlap with annotation (gene or TE) of up to 500bp 5' or 3' of the annotation unit.

Supplementary Data 9. Pollen-genes DMRs Transcript-levels. CSV file with genes directly or potentially implicated in pollen function and their references as well as DMR detection in the comparison between wild-type Col-0 VC and SC or mutant VCs. RNA-seq data from pollen and seedling are included as well as qRT-PCR results performed in this study.

Supplementary Data 10. Pollen-genes RTqPCR. CSV file of all genes shown in figure 4b providing transcript levels for all biological replicates of wild-type (Col-0) and mutant pollen as well as values of Student T-test evaluation for the comparison between mutant and wild type transcript levels.

Supplementary Data 11. Primer-sequences. Excel file containing all Primer sequences used in this study for (1) genotyping; (2) qRT-PCR; (3) construction-cloning.
