# Supplementary material for: Male fertility in Arabidopsis requires active DNA demethylation of genes that control pollen tube function
Source: Nat Commun. 2021 Jan 18;12:410. doi: 10.1038/s41467-020-20606-1 (PMC7813888; doi:10.1038/s41467-020-20606-1)
Supplement: Supplementary file 2 — Peer Review File [file 41467_2020_20606_MOESM2_ESM.pdf]

Reviewer #1 (Remarks to the Author):

Khouider et al., report the interesting discovery on the role of DNA glycosylases DEMETER (DME) and REPRESSOR OF SILENCING 1 (ROS1) in semi-redundantly in regulating the demethylation of genome DNA in the vegetative cell of pollen which contributes to proper pollen tube progression. In addition, the authors identify that four pollen-specific genes have increased DNA methylation and reduced expression levels in the single mutant dme and dme;ros1. The functional importance of these four genes is supported by the functional recovery of male fertility by over-expressing analysis. This paper is well deigned and prepared. This finding uncovers a critical gene regulatory function of active DNA demethylation in regulating pollen function and male fertility.

Comments:
1)In page 7 line 164-167, the authors described six genes with reduced transcript levels in mutant pollen, why four genes were selected for ectopic expression analysis in page 9.
2)To convince the regulatory role of active DNA demethylation on the four genes by DME and ROS1, I suggest the authors to show more developmental and genetic evidence of these four genes such as the mutant analysis and expression assay in pollen vegetative cells and sperms.

Reviewer #2 (Remarks to the Author):

The authors address an important biology question: how active DNA demethylation regulate gene expression to control pollen function and male fertility. The authors showed that DME and ROS1 is required for the DNA demethylation and activation of genes involved in pollen tube function. This provided a molecular explanation for the male sterility phenotypes

The manuscript is overall well-written and well-explained supplementary material; however, there are additional considerations can be included in the manuscript to improve the reproducibility and robustness of the analysis.

Major revisions:

1. In the section of "Whole genome bisulfite sequencing and DMR calling", what criteria or statistical method were used to define "differentially methylated mCGs, mCHGs or mCHHs". Did you apply "an absolute methylation 336 difference of 0.35" to all three contexts?

2. How "genes associated with such CG hypermethylation" were defined at Line 139? How GO enrichment was performed? What background was used for GO enrichment analysis? Did the authors use all genes that were covered by the methylome data or simply genes on the genome? The details of the methods should be documented. Moreover, it's not clear which comparison the authors used here. It would be nice to provide the list of gene in a table.

3. In Fig2A, how TE, intergenic and genes were assigned to DMRs? This should be documented in the method section.

4. A heatmap should be generated to show the differences of DNA methylation patterns in wild type plants and mutant plants using the data from Fig2C.

5. In Line 152, the authors mentioned that they did "Visual inspection of our 153 methylome data revealed higher DNA methylation for 74 genes". The authors should specify what criteria they used for visual inspection.

6. In the 74 genes, the author should include information about: 1) for the regions highlighted in the

figure, whether they were identified as DMR using the method documented by the author; if not, what's the percentage of regions that can be detected as DMR. 2) whether there was any part of the highlighted regions annotated as TE/repeat. If so, what was the percentage?

7. The authors should provide results to show that in transgenic lines gene expression was re re-established.

Reviewer #3 (Remarks to the Author):

In this work, Khouider et al., investigate the role of two enzymes responsible for active DNA demethylation in the pollen, namely DME and ROS1. Defects of paternal transmission of dme/+ were previously reported in Schoft et al 2011, however the reasons for this defect were only partially answered. Here, the authors found that low paternal transmission of dme/+ mutant can be worsened in a dme/+ ros1 double mutant indicating a redundancy between those two genes. Additionally, they further demonstrate this redundancy at the molecular level using GWBS. The authors show that DNA demethylation of a few genes in the pollen vegetative cell is required for their proper expression and that their miss-regulation can partially complement the dme/+ and dme/+ros1 paternal transmission defect. This key result suggests that beyond a role in transposable element control, DNA demethylation of the vegetative cell is involved in the regulation of developmental genes required for proper pollen function.
In this work, the results are interesting and important, and will certainly advance our understanding of the importance of vegetative cell demethylation for pollen development. However, some parts of the results have flaws that need to be address before publication.

Major points

• The authors need to better characterize the phenotype. They should provide some quantification of the phenotype (eg measurement of the width of the aniline blue staining at maybe two position along the tract in a few independent siliques or maybe at different time point after pollination) such that the reader can evaluate the strength and frequency of the phenotype. The authors state that the pollen tubes fail to orient properly but they do not provide a quantification corresponding to this statement (the authors could for example measure the angle in-between the pollen tube and the septum).
• The authors should check pollen germination as it was previously determined by Schoft et al to be involved in the defect of paternal transmission of the dme/+ mutant. Additionally, considering the genes identified by the authors, it would be very interesting if the authors could check if the mutant pollen show defect in pollen tube guidance.
• All transmission analyses are done on only one allele and by using sulfadiazine resistance. The authors should, at least for figure 1, redo the analysis with additional dme alleles. Considering the small variation in percentage observed by the authors in Fig1C and Fig4B, the authors should also confirm, with an equal n number, that they obtain similar percentage between genotyping and sulfadiazine resistance. For the same allele, dme-6/+, Schoft et al observed 21.5 % paternal transmission (n=214) but the authors here observed 17.45% (n=2833).
• To strengthen their conclusion concerning the post meiotic effect of the mutation, the authors should complement dme/+ transmission defect using a DME transgene expressed post meiotically such as under pLAT52, pKRP6 or pVCK1.
• The authors should assess the pollen progression phenotype in mutants affecting the candidate genes PLOU, PRK4, PRK2 and RKF2 and in the complemented lines of Fig 4 B otherwise they cannot make the link between the candidate genes, the defect in transmission and the pollen progression phenotype.

Minor points

- The authors should discuss why the transmission does not go back to 50% even in case of ovule excess as it suggests the presence additional phenotype.
- In Figure 1B, It would be good to have the autofluorescence on both view.
- In Figure 3: add p values on qPCR
- The author should provide more information on how they performed the experiment with limited pollen.
- Optional: Do the authors see a similar phenotype or modification of dme/+ phenotype using mutants affecting the two other DNA glycosylase, dml2 and dml3.
- The authors should tone down: "dramatically" line107, "sharp" line 195 and "key genes necessary for pollen tube progression" line 206.

Point by point response to the reviewers' comments

We would like first to thank the reviewers for their interest in our work and their criticisms, which helped us to produce a stronger and more complete manuscript. We apologize for the long delay in producing the revised manuscript, which was due to the complete shutdown of our laboratory for over two months and the very limited access we had to it for another month. All changes made in the manuscript are labeled.

Reviewer #1 (Remarks to the Author):
Khouider et al., report the interesting discovery on the role of DNA glycosylases DEMETER (DME) and REPRESSOR OF SILENCING 1 (ROS1) in semi-redundantly in regulating the demethylation of genome DNA in the vegetative cell of pollen which contributes to proper pollen tube progression. In addition, the authors identify that four pollen-specific genes have increased DNA methylation and reduced expression levels in the single mutant dme and dme;ros1. The functional importance of these four genes is supported by the functional recovery of male fertility by over-expressing analysis. This paper is well deigned and prepared. This finding uncovers a critical gene regulatory function of active DNA demethylation in regulating pollen function and male fertility.

Comments:
1)In page 7 line 164-167, the authors described six genes with reduced transcript levels in mutant pollen, why four genes were selected for ectopic expression analysis in page 9.

We actually selected all six genes with reduced transcript levels in mutant pollen for the complementation approach (Figure 4). Four of these six genes show partial restoration of fertility in *dme/+;ros1*, and three of these in *dme/+* mutants.

2)To convince the regulatory role of active DNA demethylation on the four genes by DME and ROS1, I suggest the authors to show more developmental and genetic evidence of these four genes such as the mutant analysis and expression assay in pollen vegetative cells and sperms.

We agree that the functional characterization of the identified demethylation targets is an important next step, but such a study is beyond the scope of the present work, which aimed at determining if DNA demethylation is implicated in the regulation of genes required for male fertility. The partial rescue of male fertility in demethylase mutant pollen obtained by restoring the expression of any of the four target genes indeed provides strong evidence for a role of these genes in pollen function. To avoid over-interpretations of our results, we now use the more general term 'pollen tube function' instead of 'pollen tube guidance' or similar, function-specific terms to describe the role of the DME/ROS1 target genes.
Previous publications indicate that single mutants of the target genes do not show fertility defects (Chang et al., Mol Plant, 2013; Takeuchi & Higashiyama, Nature 2016). We have begun the generation of multiple mutant combinations but this work is far from being complete and it will serve as the basis for follow-up analyses of the four target genes. Of note, there are no mutant lines for *AT2G16030/PLOU*, which means that we have to resort to the CRISPR/Cas9 approach to generate one.

Reviewer #2 (Remarks to the Author):

The authors address an important biology question: how active DNA demethylation regulate gene expression to control pollen function and male fertility. The authors showed that DME and ROS1 is required for the DNA demethylation and activation of genes involved in pollen tube function. This provided a molecular explanation for the male sterility phenotypes

The manuscript is overall well-written and well-explained supplementary material; however, there are additional considerations can be included in the manuscript to improve the reproducibility and robustness of the analysis.

Major revisions:

1. In the section of "Whole genome bisulfite sequencing and DMR calling", what criteria or statistical method were used to define "differentially methylated mCGs, mCHGs or mCHHs". Did you apply "an absolute methylation 336 difference of 0.35" to all three contexts?

Based on the reviewer's comment, we chose to reinvestigate the methylome data in more detail. This led us to change the DMR-calling criteria to more 'standard' values, taking into account the different levels of methylation for the respective contexts (see Stroud et al, Cell 2013). Also, we took into consideration this time the fact that ectopic DNA methylation in mutant vegetative cells is frequently confined to short fragments, very often located in the upstream/promoter region of the proximal gene. Thus, DMRs are now defined as 50-bp bins containing at least 2, 3 or 4 differentially methylated mCGs, mCHGs or mCHHs with an absolute methylation difference of 0.40, 0.25 and 0.10, respectively (bins localizing within 400 bp of each other were merged). Moreover, we now have included annotations that associate with DMRs within 500bp 5' and 3' of annotation units (TAIR10). Although this led to an increase in detecting hypo- and hypermethylated DMRs, our initial findings that CG-hypomethylation diminishes in *dme/+* and *dme/+;ros1-* derived vegetative cells (VCs) versus sperm cells (SCs) has not changed (Figure S2A). In addition, we now show that the size of the CG-hypo DMRs detected in VCs versus SCs is decreasing strongly in mutants compared to wild type (Figure S2C). Importantly, protein coding genes associated with CG-hypo DMRs in the VC are enriched for Gene ontology categories that point towards a direct role in pollen tube function, especially signaling components (Table 1). We have not applied statistical measures for the detection of DMRs as the overall coverage of the different WGBS samples is rather low, due to the limited amount of material. Nonetheless, further analysis, such as heat-maps of absolute DNA methylation levels in SCs and VCs clearly show that the VC hypomethylation in wild type VCs is dependent on the demethylases (Figure 2C) and CG-hypo DMRs detected between wild-type and mutant VCs strongly overlap with the CG-hypo DMRs detected between VCs and SCs in wild type pollen (Figure S2E,F). In summary, we provide a much more detailed and robust analysis of methylome data, which reinforces and expands the conclusions of our initial analysis (see also next point).

2. How "genes associated with such CG hypermethylation" were defined at Line 139? How GO enrichment was performed? What background was used for GO enrichment analysis? Did the authors use all genes that were covered by the methylome data or simply genes on the genome? The details of the methods should be documented. Moreover, it's not clear which comparison the authors used here. It would be nice to provide the list of gene in a table.

Based on the novel DMR-calling criteria (see point 1), we were now able to associate DMRs to more than half of the genes involved in pollen function in the list we have generated based on literature research (Table S9 and Figure 3A). Using the DAVID functional gene annotation tool, with the *Arabidopsis thaliana* TAIR10 genome as background, we now find Gene Ontology enrichments in pollen tube function as well as categories related to signal transduction (see Table 1). We have documented the GO-term analysis in the Methods section. Annotations (protein coding genes and TEs) associated with DMRs are included in the DMR tables, listing DMRs detected in the different comparisons (Table S2-S8).

3. In Fig2A, how TE, intergenic and genes were assigned to DMRs? This should be documented in the method section.

DMRs that overlap or are found within 500bp 5' and 3' of the annotation unit are associated with it. Genes that overlap with TEs within this 500bp 5' and 3' of their annotation units are now categorized as 'Overlap Genes-TEs'. Only DMRs that do not overlap within 500bp up- or downstream of the annotation are called 'intergenic'. This is now also described in the Methods section.

4. A heatmap should be generated to show the differences of DNA methylation patterns in wild type plants and mutant plants using the data from Fig2C.

We have replaced the box-plot of the absolute CG methylation level in SCs and VCs by a heat map, showing the absolute CG methylation level for each DMR and genotype for SCs and VCs, respectively (Figure 2C). We have also included a heat-map, based on the CG-hypo DMRs detected between wild type and *dme/+;ros1* VCs (Figure S2F).

5. In Line 152, the authors mentioned that they did "Visual inspection of our 153 methylome data revealed higher DNA methylation for 74 genes". The authors should specify what criteria they used for visual inspection.

We have now directly compared the list of DMR-associated genes with the list of genes involved in pollen tube function. In total 101 of these 196 genes show hypo DNA methylation either in the comparison between wild-type VCs vs. SCs or between wild-type vs. mutant VCs (Figure 3A; Table S9).

6. In the 74 genes, the author should include information about: 1) for the regions highlighted in the figure, whether they were identified as DMR using the method documented by the author; if not, what's the percentage of regions that can be detected as DMR. 2) whether there was any part of the highlighted regions annotated as TE/repeat. If so, what was the percentage?

1) All loci shown in Figure S3 are now included in the DMR-associated annotations list (Table S9).
2) We have now included this information in Table S9. In general 60% of the DMR-associated genes overlap with a TE-sequence within 500bp 5' or 3' of the annotation unit. This is similar to the pollen-function genes that show DNA hypomethylation in wild-type VCs versus SCs (24/43 genes = 56%).

7. The authors should provide results to show that in transgenic lines gene expression was re re-established.

We have performed RT-qPCR experiments for all of the transgenes carrying target genes in the *dme/+* background and found that in all cases the transgenes are expressed (Table S10).

Reviewer #3 (Remarks to the Author):

In this work, Khouider et al., investigate the role of two enzymes responsible for active DNA demethylation in the pollen, namely DME and ROS1. Defects of paternal transmission of dme/+ were previously reported in Schoft et al 2011, however the reasons for this defect were only partially answered. Here, the authors found that low paternal transmission of dme/+ mutant can be worsened in a dme/+ ros1 double mutant indicating a redundancy between those two genes. Additionally, they further demonstrate this redundancy at the molecular level using GWBS. The authors show that DNA demethylation of a few genes in the pollen vegetative cell is required for their proper expression and that their miss-regulation can partially complement the dme/+ and dme/+ros1 paternal transmission defect. This key result suggests that beyond a role in transposable element control, DNA demethylation of the vegetative cell is involved in the regulation of developmental genes required for proper pollen function.
In this work, the results are interesting and important, and will certainly advance our understanding of the importance of vegetative cell demethylation for pollen development. However, some parts of the results have flaws that need to be address before publication.

Major points

• The authors need to better characterize the phenotype. They should provide some quantification of the phenotype (eg measurement of the width of the aniline blue staining at maybe two position along the tract in a few independent siliques or maybe at different time point after pollination) such that the reader can evaluate the strength and frequency of the phenotype. The authors state that the pollen tubes fail to orient properly but they do not provide a quantification corresponding to this statement (the authors could for example measure the angle in-between the pollen tube and the septum).

We agree that an in-depth characterization of the pollen phenotype is an important next step, but such a characterization is beyond the scope of the present work. Here, we provide evidence that pollen tube function requires the active demethylation of genes that form a potential signaling network. We quantify the sterility phenotype by transmission rates of the *dme* mutant allele in crosses with mutant fathers. We also made sure that seed set for each silique was not effected in these crosses to exclude the possibility of an erratic maternal effect.

• The authors should check pollen germination as it was previously determined by Schoft et al to be involved in the defect of paternal transmission of the dme/+ mutant. Additionally,

considering the genes identified by the authors, it would be very interesting if the authors could check if the mutant pollen show defect in pollen tube guidance.

Pollen germination was previously shown to contribute only partially to the fertility defect in *dme* mutants in the cited publication (Schoft et al. PNAS 2011). Therefore we investigated if pollen tube progression is affected in the mutant and indeed found a strong deviation from the linear growth pattern in *dme* and *dme;ros1* pollen. We agree that the further elucidation of the pollen tube defect in the demethylase mutants is important, but again such a study is beyond the scope of our present work. The current manuscript that focuses on the role of active DNA demethylation for regulation of genes implicated in pollen tube function. To avoid over-interpretations of our results, we now use the term 'pollen tube function' instead of 'pollen tube guidance' to describe the role of the DME/ROS1 target genes.

• *All transmission analyses are done on only one allele and by using sulfadiazine resistance. The authors should, at least for figure 1, redo the analysis with additional dme alleles. Considering the small variation in percentage observed by the authors in Fig1C and Fig4B, the authors should also confirm, with an equal n number, that they obtain similar percentage between genotyping and sulfadiazine resistance. For the same allele, dme-6/+, Schoft et al observed 21.5 % paternal transmission (n=214) but the authors here observed 17.45% (n=2833).*

Schoft et al. 2011 have tested several *dme* alleles and observed transmission deficiencies for all alleles in the Col-0 background. We have observed that this fertility defect depends to quite some extent on the respective growth conditions, especially humidity and temperature. Therefore, we did not expect to obtain identical numbers as in the publication of Schoft et al. However, we corroborated the *dme* mutant allele transmission rates obtained by antibiotic resistance with that obtained by PCR-genotyping and found almost identical number (17.5% vs. 17.8%; see Material and Methods section). Therefore we are confident that our results are sufficiently robust to support our conclusions.

• *To strengthen their conclusion concerning the post meiotic effect of the mutation, the authors should complement dme/+ transmission defect using a DME transgene expressed post meiotically such as under pLAT52, pKRP6 or pVCK1.*

We did not observe any deviation of the post-meiotic male tetrad production using the *quartet* mutant in addition to the equal distribution of pollen carrying mutant and wild type *DME* alleles in *dme/+* and *dme/+;ros1* plants. Therefore, we conclude that the demethylase mutants are devoid of any meiotic problems.
The establishment and functional analysis of lines such as those suggested by the reviewer would require several more months and cannot be done in the framework of the present study. Also, we feel that the generation of such transgenic lines expressing *DME* in a pollen-specific way would only partially answer the question about a potential meiotic function as it is impossible to generate sporophytic homozygous *dme* mutants.

• *The authors should assess the pollen progression phenotype in mutants affecting the candidate genes PLOU, PRK4, PRK2 and RKF2 and in the complemented lines of Fig 4 B otherwise they cannot make the link between the candidate genes, the defect in transmission and the pollen progression phenotype.*

Our response is the same as to reviewer 1 similar comment: We agree that the functional characterization of the identified demethylation targets is an important next step, but such a study is beyond the scope of the present work, which aimed at determining if DNA demethylation is implicated in the regulation of genes required for male fertility. The partial rescue of male fertility in demethylase mutant pollen obtained by restoring the expression of any the four target genes indeed provides strong evidence for a role of these genes in pollen function. To avoid over-interpretations of our results, we now use the term 'pollen tube function' instead of 'pollen tube guidance' to describe the role of the DME/ROS1 target genes.
Previous publications indicate that single mutants of the target genes do not show fertility defects (Chang et al., Mol Plant, 2013; Takeuchi & Higashiyama, Nature 2016). We have begun the generation of multiple mutant combinations but this work is far from being complete and it will serve as the basis for follow-up analyses of the four target genes. Of note, there are no mutant lines for *AT2G16030/PLOU*, which means that we have to resort to the CRISPR/Cas9 approach to generate one.

Minor points

• *The authors should discuss why the transmission does not go back to 50% even in case of ovule excess as it suggests the presence additional phenotype.*

We think the failure of demethylase mutant pollen to fertilize ovules even when these are over-abundant, indicates that the pollen tubes are unable to target ovules properly. This in turn strongly suggests that the mutant pollen is indeed defective in guidance signaling and not just pollen tube growth. However, as we do not have further functional evidence to underline this statement we did not further discuss this point.

• *In Figure 1B, It would be good to have the autofluorescence on both view.*

We now provide both channels in addition to the overlay in Figure 1B.

• *In Figure 3: add p values on qPCR*

We have included the p-values using student t-test in the graph (** <0.01; ***<0.001)

• *The author should provide more information on how they performed the experiment with limited pollen.*

We now present a more detailed description of the experimental design in the Methods section.

• *Optional: Do the authors see a similar phenotype or modification of dme/+ phenotype using mutants affecting the two other DNA glycosylase, dml2 and dml3.*

We have done combinations *dme* with *dml2* and *dml3* and did not observe a significant reduction in *dme* transmission. Moreover, a *dme/+;ros1;dml3* triple mutant did not show

differences in *dme* transmission rates compared to the *dme/+;ros1* double mutant. Therefore, we conclude that DME and ROS1 are the main DNA glycosylases/lyases in pollen. However, because we do not have yet the quadruple mutant, we prefer to not mention these results in the current manuscript.

• *The authors should tone down: "dramatically" line107, "sharp" line 195 and "key genes necessary for pollen tube progression" line 206.*

We have made the following changes: ‘dramatically’ -> ‘significantly’ (Line 107); ‘sharp fertility increase’ -> ‘notable fertility increase’ (Line 217); ‘key genes’ -> we have deleted the phrase.

Reviewer #1 (Remarks to the Author):

This revision addresses my concerns, and I have no further comments.

[Editor: Reviewer #2 states in Comment to Editor section that (s)he is satisfied with the revision.]

Reviewer #3 (Remarks to the Author):

The authors have addressed most of the concerns I had with the previous submission.
